# Aspiration dynamics generate robust predictions in heterogeneous populations

**DOI:** 10.1038/s41467-021-23548-4

**Published:** 2021-05-31

**Authors:** Lei Zhou, Bin Wu, Jinming Du, Long Wang

**Affiliations:** 1 Center for Systems and Control, College of Engineering, Peking University, Beijing, China; 2 Center for Research on Intelligent System and Engineering, Institute of Automation, Chinese Academy of Sciences, Beijing, China; 3 School of Sciences, Beijing University of Posts and Telecommunications, Beijing, China; 4 Institute of Industrial and Systems Engineering, College of Information Science and Engineering, Northeastern University, Shenyang, China; 5 Key Laboratory of Data Analytics and Optimization for Smart Industry (Northeastern University), Ministry of Education, Shenyang, China

**Keywords:** Evolutionary theory, Social evolution, Applied mathematics, Complex networks

## Abstract

Update rules, which describe how individuals adjust their behavior over time, affect the outcome of social interactions. Theoretical studies have shown that evolutionary outcomes are sensitive to model details when update rules are imitation-based but are robust when update rules are self-evaluation based. However, studies of self-evaluation based rules have focused on homogeneous population structures where each individual has the same number of neighbors. Here, we consider heterogeneous population structures represented by weighted networks. Under weak selection, we analytically derive the condition for strategy success, which coincides with the classical condition of risk-dominance. This condition holds for all weighted networks and distributions of aspiration levels, and for individualized ways of self-evaluation. Our findings recover previous results as special cases and demonstrate the universality of the robustness property under self-evaluation based rules. Our work thus sheds light on the intrinsic difference between evolutionary dynamics under self-evaluation based and imitation-based update rules.

## Abstract (web summary)

Social interaction outcomes can depend on the type of information individuals possess and how it is used in decision-making. Here, Zhou et al. find that self-evaluation based decision-making rules lead to evolutionary outcomes that are robust to different population structures and ways of self-evaluation.

## Introduction

Social behaviors such as cooperation are deeply rooted in the daily interactions across all levels of organisms. In particular, humans exhibit remarkable capabilities of cooperating, coordinating, and dividing tasks with other humans. Such social behaviors are indispensable to the survival and development of human societies. Understanding the evolution of social behavior is thus of great importance^1,2^. After decades of investigations, evolutionary game theory has been demonstrated as a powerful tool to study the evolution of social behavior^3,4^.

Indeed, past decades have seen intensive investigations of evolutionary games in structured populations^5–9^. One of the most important questions is how population structure alters evolutionary outcomes. It is shown that the answer to this question strongly depends on update rules^7,10–12^. Update rules are explicit behavioral rules of individuals, which specify what kind of information they use and how they process such information to determine future behaviors^7^. In evolutionary games, the information required by update rules usually includes individuals’ strategies and payoffs. As the input for decision making, the information used is likely to affect individuals’ behavioral updating, resulting in changes at the population level. One kind of information that receives particular attention is social peers’ payoff information. Recent human behavioral experiments suggest that whether individuals use social peers’ payoff information to update behavior may be crucial to the outcome of social interactions, for example, the level of cooperation in groups^13,14^ or on network-structured populations^15–18^.

Based on the relevance of social peers’ payoffs, update rules in theoretical models can be classified into two classes: imitation-based (relevant) and self-evaluation based (irrelevant). Under imitation-based rules, individuals update strategies by copying more successful peers. When using self-evaluation based rules, individuals self-assess performance of strategies and then switch to strategy alternatives^7^. Self-evaluation can be based on aspirations: individuals compare payoffs with their aspirations and then switch based on the shortfall of payoffs^19–23^. Update rules of these two classes are both common in practice and they are tailored for different environment. For example, if individuals are not confident to make decisions or uncertain about the consequences, imitating the more successful provides valuable shortcuts for decision-makers. Self-evaluation, instead, is efficient and superior when social information is unavailable, regarded as unreliable, or costs individuals too much to gather and process.

It is well known that imitation-based update rules lead to evolutionary outcomes sensitive to model details, such as population structures^11,12,24^, the way of imitating^10,25^, and heterogeneity of decision-making rules^26,27^. Such sensitivity makes it difficult for researchers to generalize predictions across different population structures (e.g., from regular to non-regular networks) or different imitation-based rules (e.g., from death-birth^28^ to pairwise comparison rules^29,30^). On the other hand, self-evaluation based rules are shown to generate robust evolutionary outcomes. For instance, the condition for strategy success on unweighted regular networks is found to be the same as that in well-mixed populations^31^, and such robustness is not affected by different distributions of aspiration values^32^ or heterogeneous ways of self-evaluation^33^. Despite the findings on unweighted regular networks^31–33^, it remains unclear whether the robustness property under self-evaluation based rules applies to heterogeneous population structures, where the number of neighbors varies from one individual to another and each individual interacts with their neighbors under different rates.

To fill this gap, we study evolutionary games under aspiration-based self-evaluation rules (for short, aspiration dynamics) on heterogeneous population structures represented by weighted networks. Under the limit of weak selection and symmetric aspirations, we analytically derive a condition for one strategy to prevail over the other, which is found to coincide with the classical condition of risk-dominance. This condition holds for any weighted network, any distribution of aspiration levels, and for any individualized ways of self-evaluation. If aspirations are differentiated by strategies, we find that the condition of risk-dominance is altered and cooperation can evolve in the Prisoner’s Dilemma game if individuals aspire more when they defect. The intuitive interpretation of our results is as follows: one strategy prevails over the other if the strategy on average brings more satisfaction to individuals than the other does. Our work thus (i) highlights that switching off from social peers’ payoff information while updating strategies has a nontrivial impact on the evolutionary outcomes, and (ii) demonstrates that the robustness property of aspiration-based self-evaluation rules is universal in heterogeneous populations.

## Results

### Population structure and games

We consider a population with fixed size *N* (*N* ≥ 2). The population structure is depicted by a static weighted graph (or network) with edge weights *w*_*ij*_ ≥ 0, where vertices represent individuals, edges indicate who interacts with whom, and weights describe the number of interactions per unit time. Self-interactions are excluded. Individuals collect edge-weighted average payoffs by playing games with their nearest neighbors^24^. The total number of interactions each individual *i* engages in is ${d}_{i}=\mathop{\sum }\nolimits_{j = 1}^{N}{w}_{ij}$ (*i* = 1, 2, ⋯ , *N*). We assume *d*_*i*_ > 0 for all *i*, which means that each individual has at least one neighbor to interact with. Visually, the graph should have neither isolated vertices nor self-loops, which are natural assumptions when studying evolutionary games on graphs. In each game, individuals play either strategy *A* or strategy *B*. The payoff matrix of the game is given by

1 $$\begin{array}{l}\quad A\ \ \ B\\ \begin{array}{l}A\\ B\end{array}\left(\begin{array}{ll}a&b\\ c&d\end{array}\right),\end{array}$$

where both players get payoff *a* if they play strategy *A* (*A*-player) and get *d* if they play strategy *B* (*B*-player); if an *A*-player encounters a *B*-player, the former obtains payoff *b* and the latter *c*. For each individual *i*, we denote *π*_*i*,*X*_ as its payoff when it uses strategy *X* (*X* = *A*, *B*).

### Aspiration dynamics

**Fig. 1:**
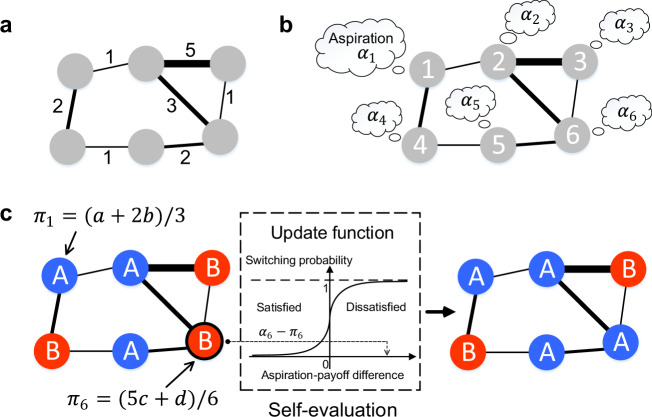
Aspiration dynamics on weighted graphs. **a** An undirected weighted graph with edge weight *w*_*ij*_ ≥ 0. **b** Individuals occupy vertices of the graph and each individual *l* has an imaginary payoff value *α*_*l*_ they aspire, called aspiration level. **c** For aspiration dynamics, at each time step, an individual is randomly selected (here, the sixth individual, marked by the black circle). It garners an edge-weighted average payoff (*π*_6_) by playing games with its nearest neighbors^24^. Then it self-evaluates the performance of the strategy in use by calculating the aspiration-payoff difference (*α*_6_ − *π*_6_), which is later used by the update function $g\!:\!{\mathbb{R}}\to [0,1]$ to determine its switching probability. If the payoff exceeds the aspiration, it feels satisfied and is more likely to keep its current strategy; otherwise, it is prone to switch. As illustrated, *α*_6_ − *π*_6_ > 0 (i.e., *π*_6_ < *α*_6_) and the corresponding individual switches from strategy *B* to *A*.

At each time step, an individual is randomly selected and given the opportunity to revise its strategy. We assume individuals follow self-evaluation-based rules, under which they evaluate their strategies by comparing payoffs garnered from the games with their aspirations. Aspirations are either personalized^32^, which means each individual *l* has its own aspiration *α*_*l*_ (*l* = 1, 2, ⋯ , *N*), or contingent on strategies, which means that individuals using strategy *A* have an aspiration *α*_*A*_ and those using *B* have *α*_*B*_. For simplicity, we consider fixed aspirations, meaning that there is no adaptation of aspirations due to learning. If such aspiration-driven update rules are deterministic, the aspiration level serves as a sharp boundary between satisfaction and disappointment^34^: if an individual’s payoff exceeds its aspiration, the outcome is deemed satisfactory and it will repeat its strategy; if the payoff is otherwise lower than the aspiration, it feels disappointed and will switch to the other strategy. In real-life situations, strategy updating involves mistakes and admits bounded rationality, which is better captured by probabilistic (stochastic) strategy switchings. The probability can be determined by the level of satisfaction, i.e., the difference between the payoff and the aspiration. In our model, to be consistent with previous work^32,33,35^, we use update functions $g\!:\!{\mathbb{R}}\to [0,1]$ to map the aspiration-payoff difference into the switching probability (see Methods). We allow individuals to have their own update functions *g*_*l*_ since they may behave differently even for the same aspiration-payoff difference (i.e., level of dissatisfaction). Albeit this flexibility, all update functions should ensure that individuals have decreasing tendency to switch a strategy if it brings more satisfaction (see Methods) (Fig. 1).

Here, we employ stochastic self-evaluation-based rules. Under stochastic rules, the strictness of the strategy evaluation, namely, how much the payoff-aspiration difference affects individuals’ decision-making, is controlled by the selection intensity *β* ≥ 0^36,37^. Since each individual on the network uses either strategy *A* or *B*, the number of all possible states of the system is *M* = 2^*N*^. Meanwhile, the transition probabilities between all the states can be described by an *M* × *M* matrix **P**_*β*_. Similar to the mutation-selection process^11,38^, the resulting aspiration dynamics admit a unique stationary distribution **u**_*β*_ (a column vector with *M* elements), which is the unique solution to the equation ${{\bf{u}}}_{\beta }^{\,\text{T}\,}{{\bf{P}}}_{\beta }={{\bf{u}}}_{\beta }^{\,\text{T}\,}$ (the superscript T represents vector/matrix transpose). In this distribution, we compare the average abundance (i.e., frequency) of strategy *A*, $\langle {x}_{A}\rangle ={{\bf{u}}}_{\beta }^{\,\text{T}\,}{\bf{x}}$, with that of *B*, $\langle {x}_{B}\rangle ={{\bf{u}}}_{\beta }^{\,\text{T}\,}({\bf{1}}-{\bf{x}})$, where **x** is the frequency of strategy *A* in each of the *M* states. If 〈*x*_*A*_〉 > 〈*x*_*B*_〉, strategy *A* prevails over *B*. Otherwise, *B* prevails over *A*. We derive the condition for strategy success, i.e., the condition which leads to 〈*x*_*A*_〉 > 〈*x*_*B*_〉 or 〈*x*_*B*_〉 > 〈*x*_*A*_〉. To make progress, we consider weak selection (i.e., 0 < *β* ≪ 1)^24,36,37,39–41^, under which individuals switch strategies with a nearly constant probability. Weak selection may arise for the following reasons: (i) individuals have no obvious preference over different strategies^42,43^, (ii) individuals are uncertain about the payoffs, aspirations, or aspiration-payoff differences due to noise or stochastic interactions^44^.

### General condition for strategy success

Given our assumptions, we calculate the average frequency difference between strategy *A* and *B*, 〈*x*_*A*_ − *x*_*B*_〉, in the stationary regime. If 〈*x*_*A*_ − *x*_*B*_〉 > 0, strategy *A* prevails over *B*; otherwise, strategy *B* prevails over *A*. At the neutral drift *β* = 0, strategy *A* and strategy *B* are of equal abundance^32^. Under weak selection 0 < *β* ≪ 1, we use perturbation theory and get that 〈*x*_*A*_ − *x*_*B*_〉 > 0 if

2 $${{\bf{u}}}_{0}^{\,\text{T}\,}{{\bf{P}}}_{0}^{\prime}{\bf{c}}\;> \; 0,$$

where ${{\bf{P}}}_{0}^{\prime}=\frac{d}{d\beta }{{\bf{P}}}_{\beta }{| }_{\beta = 0}$ and ${\bf{c}}=\mathop{\sum }\nolimits_{k = 0}^{\infty }{{\bf{P}}}_{0}^{k}(2{\bf{x}}-{\bf{1}})$ is the accumulated average abundance difference during the whole evolution at the neutral drift (see detailed calculations in Methods). In fact, condition (2) holds for a large class of evolutionary dynamics which admit a unique limiting stationary distribution and an equal abundance of strategy *A* and *B* at the neutral drift. For example, the death-birth, birth-death, and pairwise comparison process with symmetric mutations all belong to this class^11,12,24,28,30,45^.

For aspiration-based update rules, individuals update their strategies independently when *β* = 0. This makes it possible for us to calculate the exact formula of **c** even if each individual uses distinct update functions. In a nutshell, we transform the calculation under the original *N*-dimensional Markov chain with 2^*N*^ states to that under *N* one-dimensional Markov chains with 2 states by virtue of the independence of strategy updating (see detailed calculations in Supplementary Note 3.2). In addition, we adapt our method for imitation-based update rules with a shared update function, which is shown to be equivalent to that in ref. ^46^. In particular, we give the condition for strategy success on any weighted graphs under pairwise comparison rules (see Supplementary Note 3.3 for detailed calculations).

In the following, we mainly focus on aspiration-based update rules and explore how symmetric or asymmetric aspirations affect the evolutionary outcomes.

### Personalized and symmetric aspirations

**Fig. 2:**
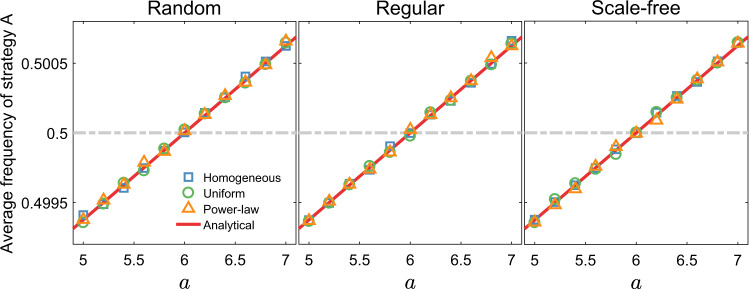
Robust predictions generated by aspiration dynamics on weighted networks. For the game, we set payoff value *b* = 0, *c* = 5, *d* = 1 and leave *a* as a tunable parameter. We plot the average frequency of strategy *A*, 〈*x*_*A*_〉, as a function of *a*. Symbols represent simulation results while solid lines are analytical ones. We construct weighted graphs by first generating an undirected graph with average degree $\bar{k}$ and then assigning weights to edges. The undirected graphs considered are random graph (left), regular graph (middle), and scale-free network (right). For each type of network, we test three edge weight distributions: homogeneous—every edge has weight one (i.e., unweighted network); uniform—edge weights are uniformly selected from the integer set {1, 2, 3, 4, 5, 6, 7, 8, 9, 10}; power-law—edge weights are randomly selected from the discrete power-law distribution (Zipf distribution with the value of the exponent equal to 3). Each data point is obtained by averaging 〈*x*_*A*_〉 in 200 independent runs. For each run, we calculate 〈*x*_*A*_〉 by averaging the frequency of strategy *A* in the last 1 × 10^7^ time steps after a transient time of 1 × 10^7^ time steps. Other parameters: *N* = 1000, $\bar{k}=6$, *α*_*l*_ = 2.0 (*l* = 1, 2, ⋯ , *N*), and *β* = 0.01.

Let us first consider personalized and symmetric aspirations. Under weak selection, we find that strategy *A* prevails over *B* if

3 $$a+b\;> \; c+d,$$

and strategy *B* prevails over *A* if *a* + *b* < *c* + *d*. This result holds for all weighted graphs without self-loops, for all distributions of aspirations, and for arbitrary number of update functions. Furthermore, if strategy *A* and *B* are both best replies to themselves (i.e., *a* > *c* and *b* < *d*), our result reduces to the classical concept of risk-dominance. It indicates that under the limit of weak selection, aspiration dynamics always select the risk-dominant strategy, which has a larger basin of attraction. In Fig. 2, we set payoff values of the game with *b* = 0, *c* = 5, and *d* = 1. Under this game, condition (3) predicts that for any weighted graphs without self-loops, *a* > 6 indicates strategy *A* prevails over *B* (equivalently, 〈*x*_*A*_〉 > 1/2); *a* < 6 leads to 〈*x*_*A*_〉 < 1/2. Our simulation results in Fig. 2 match the theoretical predictions perfectly.

The above condition significantly generalizes previous results: the selection of risk-dominant strategy on unweighted regular graphs (where all the individuals have the same number of neighbors and each individual interacts with their neighbors under identical rates)^31–33^ is generalized to non-regular and weighted graphs with individualized update functions and personalized aspirations.

For an intuitive understanding of our result, we offer the following explanations. Under weak selection, the expected payoffs of playing strategy *A* and *B* are evaluated at the neutral (*β* = 0) stationary distribution of the aspiration dynamics (see Methods for details). In this distribution, individuals update strategies independently, which makes their strategies uncorrelated. Individual *l* thus on average interacts with neighbors using strategy *A* as many times as those using *B*. This means that the expected payoffs of *l* are *π*_*l*,*A*_ = (1/2)(*a* + *b*) and *π*_*l*,*B*_ = (1/2)(*c* + *d*) when it plays strategy *A* and *B*, respectively. If *π*_*l*,*A*_ > *π*_*l*,*B*_, individual *l* is more satisfied when it uses strategy *A* and the switching rate from *A* to *B* is less than that from *B* to *A*. Note that *π*_*l*,*A*_ > *π*_*l*,*B*_ is equivalent to *a* + *b* > *c* + *d*. Therefore, individual *l* is more likely to be an *A*-player if *a* + *b* > *c* + *d*. Since the above logic applies to any individual, the condition *a* + *b* > *c* + *d* actually makes all the individuals feel more satisfied when they play strategy *A*. As a consequence, the average frequency of *A*-players in the population is greater than that of *B*-players. Similarly, *a* + *b* < *c* + *d* results in more satisfaction when individuals play strategy *B*, which makes the average frequency of *A*-players less than that of *B*-players.

Comparing our result with that in well-mixed populations (equivalent to a complete graph in our model)^31^, we show that population structure does not alter the condition for strategy success. In other words, the condition for strategy success under aspiration dynamics is robust to the underlying population structure. The robustness property has practical advantages on strategy selection^11^: (i) for a fixed game, the predictions are the same for a large class of population structures; (ii) to tell which strategy succeeds, the population can be assumed to be well-mixed.

**Fig. 3:**
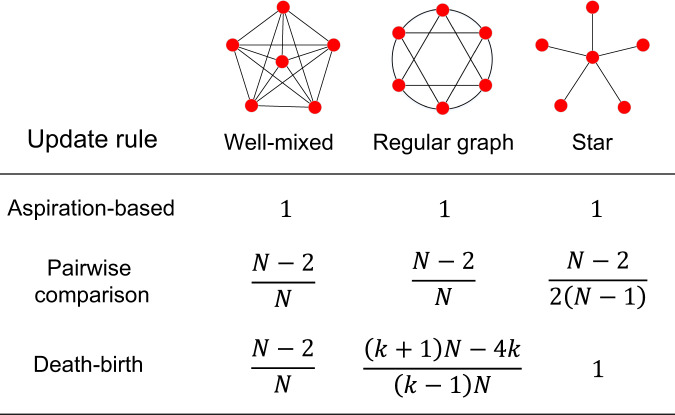
The structure coefficients *σ* for aspiration-based and imitation-based update rules. The two imitation-based rules shown here are pairwise comparison^29,30^ and death-birth update rule^28^. Here, the parameter *N* represents the population size and *k* the degree of the regular graph (i.e., the number of neighbors each individual has). For strategy *A* to be favored over strategy *B*, the structure coefficient *σ* can be interpreted as the required degree of assortment among individuals who use the same strategy^11^. All the *σ*s in the table are obtained under the limit of weak selection. In addition, the limit of rare mutation is assumed under imitation-based rules. We also derive a general formula under pairwise comparison update rules for any weighted graphs in Supplementary Note 3.3.

Besides, by generalizing the main Theorem in ref. ^32^ to non-regular graphs, our result is related to the structure coefficient *σ*^8,11^ derived for weak selection. It is shown that *σ* depends on the update rule and the population structure (including the population size *N*). But it does not depend on the payoff entries. It summarizes the effect of population structure on the condition for strategy success. Intuitively, for strategy *A* to be favored over strategy *B*, *σ* quantifies the required degree of assortment among individuals who use the same strategy^11^, and *σ* > 1 implies that individuals with the same strategy are more likely to interact with each other than those with different strategies. For aspiration dynamics, we prove that *σ* = 1 for a large class of population structures and there is no dependence on the population size. This contrasts with the result obtained under imitation-based rules, which are shown to sensitively depend on the population structure and the population size^11,24^. To better illustrate the difference between aspiration-based and imitation-based rules, we plot Fig. 3 to compare the structure coefficients on three common graphs. Here, *σ* = 1 indicates that self-evaluation-based rules do not lead to assortment of strategies for the purpose of strategy selection.

**Fig. 4:**
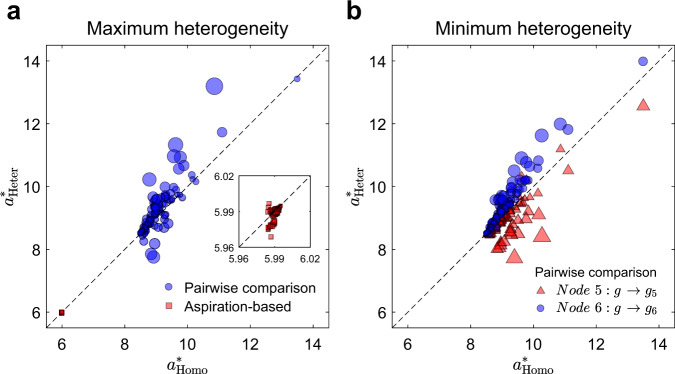
Robustness of aspiration dynamics vs. sensitivity of imitative dynamics to heterogeneity of decision-making functions for all networks of size six (*N* = 6). There are 112 connected and unweighted networks of size six. For each network, we calculate the critical value of *a*, a^*^, above which 〈*x*_*A*_〉 > 〈*x*_*B*_〉 and below which 〈*x*_*A*_〉 < 〈*x*_*B*_〉. The critical value a^*^ under two populations are calculated: (i) ${a}_{{\rm{Homo}}}^{* }$, a homogeneous population where everyone shares the same decision-making function *g*(*u*); (ii) ${a}_{{\rm{Heter}}}^{* }$, a heterogeneous population where each individual has its own decision-making functions, i.e., individual *i* uses *g*_*i*_(*u*). In panels (**a**) and (**b**), each symbol represents a pair of critical values $({a}_{{\rm{Homo}}}^{* },{a}_{{\rm{Heter}}}^{* })$ calculated under one of the networks. The size of the symbols indicates how much the heterogeneity of decision-making functions affects the evolutionary outcomes, which is proportional to the absolute value of ${a}_{{\rm{Heter}}}^{* }-{a}_{{\rm{Homo}}}^{* }$. The larger the size of the symbol, the more sensitive the evolutionary outcome to individual heterogeneity. In panel **a**, we consider maximum heterogeneity of decision-making functions (i.e., for any *i* ≠ *j*, *g*_*i*_ ≠ *g*_*j*_) under both aspiration-based (red squares) and imitation-based (blue circles) rules. In panel **b**, we consider the minimum heterogeneity under imitation-based rules, where all the individuals share the same decision-making function *g*(*u*) except one. Here, the blue circles are the results obtained when *g*_6_ ≠ *g*, and the red triangles are the results when *g*_5_ ≠ *g*. In all the calculations, we set $g(u)={g}_{1}(u)=1/(1+\exp (-u))$, ${g}_{2}(u)=(1+{\rm{erf}}(u))/2$, ${g}_{3}(u)=(1+\tanh (u))/2$, ${g}_{4}(u)=1/(1+\exp (-u/2))$, ${g}_{5}(u)=1/(1+10\exp (-u))$, and ${g}_{6}(u)=10/(10+\exp (-u))$. Other parameters: *b* = 0, *c* = 5, *d* = 1, *α*_*l*_ = 1.0 (*l* = 1, 2, ⋯ , *N*), *β* = 0.01, and *μ* → 0 (see Supplementary Note 3.3 for definitions).

In the meanwhile, we show that the results under imitation-based rules are also sensitive to the heterogeneity of update functions, even if we consider minimum heterogeneity (see Fig. 4b and Supplementary Fig. 1). In contrast, maximum heterogeneity of update functions does not alter the evolutionary outcomes induced by aspiration-based rules (see Fig. 4a). Such results demonstrate that aspiration-based rules lead to evolutionary outcomes robust to individual heterogeneities while imitation-based rules show great sensitivity.

### Asymmetric aspirations contingent on strategies

When individuals’ aspirations are contingent on their strategy in use (and thereby asymmetric), we find that the condition for strategy *A* to be favored over *B* under weak selection is $a+b\;> \;c+d-2\left({\alpha }_{B}-{\alpha }_{A}\right)$, where *α*_*X*_ is the aspiration of *X*-players (*X* = *A*, *B*). Note that the condition now depends on aspirations. Nonetheless, it is still robust to population structures, which generates invariant predictions for a large class of population structures. Intuitively, the symmetry breaking of aspiration levels leads to additional asymmetry between strategy *A* and *B*: *A*-players not only gain a different payoff but also have a different benchmark for satisfaction from *B*-players. The expected level of satisfaction is now modified as (1/2)(*a* + *b*) − *α*_*A*_ and (1/2)(*c* + *d*) − *α*_*B*_ for *A*-players and *B*-players, respectively. This modification alters the condition of risk-dominance derived under personalized aspirations and results in the dependence on aspiration levels.

So far, we only consider average payoffs. Our framework also applies to accumulated payoffs (see Supplementary Note 3.2.4 for the condition for strategy success). We show that the condition of risk-dominance is invariant under accumulated payoffs, provided that aspirations are not contingent on strategies and the selection intensity is weak. In addition, we also verify the condition for strategy success (i.e., inequality (3)) under the classical payoff scheme of *R* = 1, *S*, *T*, *P* = 0 (see Supplementary Fig. 2), and confirm the robustness of aspiration dynamics as well as the sensitivity of imitative dynamics to the heterogeneity of decision-making functions on large networks (see Supplementary Fig. 3).

## Discussion

In this work, we present a general framework to study aspiration dynamics in heterogeneous populations, which makes it possible to study the joint effect of heterogeneous population structure, personalized aspiration values, and individualized update rules on the evolutionary outcomes. Previous studies^23,31–33^, due to the limitation of their methodology, can only handle regular graphs. Under our framework, we show that under weak selection, the condition for one strategy to be selected over the other is invariant on different population structures and under various kinds of individual heterogeneities. Moreover, this condition coincides with the condition of risk-dominance. It indicates that aspiration dynamics always select the risk-dominant strategy. When individuals’ aspirations are contingent on strategies and thus asymmetric, the condition for strategy success is altered and determined by the difference between the aspirations of distinct strategies. In this case, cooperation can evolve in the Prisoner’s Dilemma if individuals aspire more when they defect.

Our framework can also be used to study imitation-based update rules. We demonstrate that our approach is equivalent to that in refs. ^24,46^ when all the individuals share the same imitation-based update function (see Supplementary Note 3.3 for details). Moreover, our results for the pairwise comparison rule show that cooperation can never evolve on any weighted networks since the critical benefit-to-cost ratio is negative and the evolutionary outcomes are greatly affected by the heterogeneity of update functions. This confirms that evolutionary outcomes induced by imitation-based rules are very sensitive to model details. Compared with the results under aspiration dynamics, it highlights the advantage of aspiration-based update rules, which generates robust evolutionary outcomes.

In a nutshell, the primary contribution of our work is three-fold: (i) we derive a general condition for strategy success that applies to a large class of evolutionary dynamics (see Supplementary Note 3 for details); (ii) we prove that for aspiration dynamics, the condition for strategy success is invariant on any weighted networks, which reveals the remarkable robustness of aspiration dynamics to the underlying population structure; (iii) the robustness property of aspiration dynamics is shown to be universal with respect to various kinds of heterogeneities and their aggregations, including heterogeneity of aspiration values, heterogeneity of update functions, and heterogeneity of social ties.

Although our work provides a general framework to study evolutionary dynamics in heterogeneous populations, theoretical results are obtained under the limit of weak selection. In this selection regime, the payoffs garnered by individuals affect minimally their probability of changing strategies. If the selection intensity becomes strong, individuals’ strategy updating will be strongly affected by payoffs and aspirations. In this case, our theoretical results may no longer apply. Despite this limitation, it is still necessary and useful to conduct theoretical analysis under weak selection since (i) it may be by far the only way to obtain analytical results for evolutionary dynamics on heterogeneous networks^24,47^, and (ii) the theoretical predictions obtained can provide guidance for future studies (e.g., testing such predictions experimentally^15–18^).

For self-evaluation-based update rules we focus on in this paper, in addition to the irrelevance of social information, they also have other features: self-evaluation-based rules are innovative^7^, which means they can revive strategies absent in the neighborhood without additional mechanisms such as random exploration or mutation; they prescribe increasing tendency to cooperate when more cooperators are present in the neighborhood for the Prisoner’s Dilemma (similar to conditional cooperators^48^). These features seem to be consistent with the recent findings on the possible features of human strategy updating^15–18^. This suggests that self-evaluation-based rules may be a good candidate for human strategy updating, which needs further empirical test.

In addition, aspiration-based self-evaluation rules are related to reinforcement learning. The rationale behind reinforcement learning is the law of effect stated by Thorndike in 1898: actions bringing satisfactory effect will be more likely to be repeated and those leading to discomfort will be less likely to occur. This is similar to our stochastic update rules, except the reinforcement of actions^49,50^. In practice, aspiration can also evolve based on past experience^22^. Although these features are not considered in our models, our work provides an important step towards multi-agent learning in heterogeneous populations, whereas literature on reinforcement learning usually focuses on the simplest two-person repeated games (see a few recent exceptions on regular graphs^51,52^). Extending our model to incorporate aspiration adaptation and reinforcement of actions is a future direction.

For the evolution of human cooperation, our work suggests the investigations of which update rules human actually uses for strategy updating. A promising direction is to conduct experiments explicitly manipulating the information availability or monitoring the information request during the game^14,53^. Then, based on the distinct informational requirements of self-evaluation-based and imitation-based rules, we may infer under what conditions human subjects tend to use these two classes of update rules and how they implement them. For theoretical studies, our work reveals a class of update rules which generate robust predictions for strategy success on a large class of population structures. The reason may lie in the irrelevance of social peers’ payoffs for strategy updating. It remains unclear what other assumptions in update rules crucially affect evolutionary outcomes. Future work along this line may lead to a deeper understanding of how update rules alter the evolutionary outcomes, which may help design the optimal decision-making rules for cooperation.

## Methods

### Notation

The population consists of *N* individuals. Each individual either uses strategy *A* or strategy *B*. We use *s*_*i*_ to denote the strategy of individual *i*: *s*_*i*_ = 1 if individual *i* uses strategy *A*, otherwise *s*_*i*_ = 0. The state of the population is represented by a column vector ${\bf{s}}={({s}_{1},{s}_{2},{s}_{3},\cdots ,{s}_{N})}^{{\rm{T}}}$ and **s** = **1** means all the individuals in the population use strategy *A*. At state **s**, we denote the frequency (i.e., abundance) of strategy *A* as ${x}_{A}({\bf{s}})=\mathop{\sum }\nolimits_{l = 1}^{N}{s}_{l}/N$ and that of strategy *B* as *x*_*B*_(**s**) = 1 − *x*_*A*_(**s**). Since each individual can use strategy *A* or strategy *B*, the number of all possible states of the population is *M* = 2^*N*^. A convenient way of indexing the state is to convert the binary vector **s** to a decimal number and plus one, which makes the index of states range from 1 to *M*. Here, we use the column vector ${\bf{x}}=\left({x}_{A}({{\bf{s}}}_{1}),{x}_{A}({{\bf{s}}}_{2}),\cdots \ ,{x}_{A}({{\bf{s}}}_{M})\right)$ to represent the frequency of strategy *A* at each state.

Meanwhile, individuals play game (1) with their neighbors and obtain edge-weighted average payoffs. For instance, at state **s**, individual *l* gets a payoff ${\pi }_{l}({\bf{s}})=\mathop{\sum }\nolimits_{k = 1}^{N}{w}_{lk}[a{s}_{l}{s}_{k}+b{s}_{l}(1-{s}_{k})+c(1-{s}_{l}){s}_{k}+d(1-{s}_{l})(1-{s}_{k})]/{d}_{l}$.

### General condition for strategy success

For evolutionary dynamics which can be modeled by an irreducible and aperiodic Markov chain, a unique limiting stationary distribution ${\bf{u}}={({u}_{1},{u}_{2},\cdots ,{u}_{j},\cdots ,{u}_{M})}^{{\rm{T}}}$ is guaranteed, where *u*_*j*_ means the probability to occur at state *j* in the stationary distribution. We denote the average frequency of strategy *A* in the stationary distribution as 〈*x*_*A*_〉 = **u**^T^**x** and that of *B* as 〈*x*_*B*_〉 = **u**^T^(**1** − **x**). In addition, a transition matrix **P** is constructed where its (*i*, *j*)-th element *p*_*ij*_ represents the probability to transit from state *i* to state *j*. Since the stationary distribution **u**, transition matrix **P**, and average frequency of strategies 〈*x*_*A*_〉, 〈*x*_*B*_〉 are determined by the selection intensity *β*, we rewrite them explicitly as **u**_*β*_, **P**_*β*_, ${\langle {x}_{A}\rangle }_{\beta }$, and ${\langle {x}_{B}\rangle }_{\beta }$. By the definition of stationary distribution, ${{\bf{u}}}_{\beta }^{{\rm{T}}}{{\bf{P}}}_{\beta }={{\bf{u}}}_{\beta }^{{\rm{T}}}$. Differentiating both sides with respect to *β* at *β* = 0 and rearranging the items, we have

4 $${({{\bf{u}}}_{0}^{\prime})}^{\text{T}}={{\bf{u}}}_{0}^{\,\text{T}\,}{{\bf{P}}}_{0}^{\prime}{\left({\bf{I}}-{{\bf{P}}}_{0}+{\bf{1}}{{\bf{u}}}_{0}^{\text{T}}\right)}^{-1}.$$

where **I** is the identity matrix of dimension *M*, ${{\bf{u}}}_{0}^{\prime}=\frac{d}{d\beta }{{\bf{u}}}_{\beta }{| }_{\beta = 0}$, and ${{\bf{P}}}_{0}^{\prime}=\frac{d}{d\beta }{{\bf{P}}}_{\beta }{| }_{\beta = 0}$.

Under weak selection *β* → 0,

5 $${\langle {x}_{A}\rangle }_{\beta }-{\langle {x}_{B}\rangle }_{\beta }={{\bf{u}}}_{0}^{\,\text{T}\,}\left(2{\bf{x}}-{\bf{1}}\right)+{\left({{\bf{u}}}_{0}^{\prime}\right)}^{\text{T}}\left(2{\bf{x}}-{\bf{1}}\right)\beta +O({\beta }^{2}).$$

Here, we assume that for the evolutionary dynamics we are focusing on, the average frequency of strategy *A* equals to that of strategy *B* at the neutral drift *β* = 0, which means that ${{\bf{u}}}_{0}^{\,\text{T}\,}\left(2{\bf{x}}-{\bf{1}}\right)=0$. Therefore, under weak selection, strategy *A* prevails over *B* (i.e., ${\langle {x}_{A}\rangle }_{\beta }> {\langle {x}_{B}\rangle }_{\beta }$) if

6 $${{\bf{u}}}_{0}^{\,\text{T}\,}{{\bf{P}}}_{0}^{\prime}\mathop{\sum }\limits_{k=0}^{\infty }{{\bf{P}}}_{0}^{k}(2{\bf{x}}-{\bf{1}})\;> \; 0,$$

and strategy *B* prevails over *A* if ${{\bf{u}}}_{0}^{\,\text{T}\,}{{\bf{P}}}_{0}^{\prime}\mathop{\sum }\nolimits_{k = 0}^{\infty }{{\bf{P}}}_{0}^{k}(2{\bf{x}}-{\bf{1}})\;<\;0$. Let ${\bf{c}}=\mathop{\sum }\nolimits_{k = 0}^{\infty }{{\bf{P}}}_{0}^{k}(2{\bf{x}}-{\bf{1}})={({c}_{1},{c}_{2},\cdots ,{c}_{M})}^{\text{T}}$, which represents the accumulated average abundance difference between strategy *A* and *B* during the whole evolution. Then, we get condition (2) in the main text.

### Condition for strategy success under aspiration dynamics

For aspiration-based update rules, we first define individual *l*’s update function as *g*_*l*_(*u*) (*l* = 1, 2, ⋯ , *N*) and these functions represent the tendency to switch strategies. Here, *u* = *β*(*α*_*l*_ − *π*_*l*_), where *α*_*l*_ is individual *l*’s aspiration level, *π*_*l*_ is its payoff, and *β* > 0 is the intensity of selection^24,36^. Weak selection means *β* ≪ 1 and *β* = 0 is the neutral drift^11^. In addition, each function *g*_*l*_(*u*) should satisfy (i) it is a probability, i.e., *g*_*l*_(*u*) ∈ [0, 1] for $u\in {\mathbb{R}}$; (ii) it is a strictly increasing function of *u*, i.e., ${g}_{l}^{\prime}(u)=d{g}_{l}(u)/du\;> \; 0$ for all *u*, which indicates that individuals with higher payoffs should have a lower tendency to switch; (iii) *g*_*l*_(0) > 0, which avoids frozen dynamics at the neutral drift.

The population dynamics governed by aspiration-based update rules can be described by an irreducible and aperiodic Markov chain^32^. Meanwhile, such dynamics also lead to an equal average abundance of strategy *A* and *B* at the neutral drift. Therefore, the general condition (2) applies to aspiration-based update rules.

To calculate the exact formula of **c** in condition (2), we utilize the fact that individuals update their strategies independently when *β* = 0 for aspiration-based update rules. This makes it possible to calculate **c** by summing up all the individual contributions. For each individual, its dynamics can be modeled by a one-dimensional Markov chain with 2 states. For instance, the transition matrix for individual *l* is

7 $$\begin{array}{l}\quad 1\quad \quad \quad \quad 0\\ \begin{array}{l}1\\ 0\end{array}\left(\begin{array}{ll}1-\frac{1}{N}{g}_{l}(0)&\frac{1}{N}{g}_{l}(0)\\ \frac{1}{N}{g}_{l}(0)&1-\frac{1}{N}{g}_{l}(0)\end{array}\right).\end{array}$$

and its individual contribution to the accumulated average abundance difference during the whole evolution is $\left(2{{\bf{s}}}_{i}(l)-1\right)/\left(2{g}_{l}(0)\right)$ where **s**_*i*_(*l*) is the strategy of individual *l* at state *i*. Based on these, we have

8 $${c}_{i}=\mathop{\sum }\limits_{l=1}^{N}\frac{2{{\bf{s}}}_{i}(l)-1}{2{g}_{l}(0)}.$$

Denote $h({{\bf{s}}}_{i})=\mathop{\sum }\nolimits_{j = 1}^{M}{p}_{ij}^{\prime}{c}_{j}=\mathop{\sum }\nolimits_{j = 1}^{M}{p}_{ij}^{\prime}\mathop{\sum }\nolimits_{l = 1}^{N}\frac{2{{\bf{s}}}_{j}(l)-1}{2{g}_{l}(0)}$. Condition (2) in the main text implies that strategy *A* prevails over *B* if

9 $${\langle h({\bf{s}})\rangle }_{0}\;> \; 0,$$

where the bracket 〈⋅〉_0_ means to take the average over the neutral stationary distribution (i.e., when *β* = 0). To evaluate 〈*h*(**s**)〉_0_, we need to know the correlation of strategies in the neutral stationary distribution, i.e., ${\langle {s}_{l}{s}_{k}\rangle }_{0}$ for any *l*, *k*. Under aspiration dynamics, at the neutral drift *β* = 0, individuals’ strategy updating does not depend on the aspiration level, the payoff, and the population structure. This implies that the transition probabilities between any two states are thus the same in both directions, which indicates ${{\bf{P}}}_{0}={{\bf{P}}}_{0}^{\,\text{T}\,}$. By the uniqueness of the stationary distribution **u**_0_ and the property of the transition (stochastic) matrix **P**_0_**1** = **1**, we have **u**_0_ = 2^−*N*^**1**. This leads to that at the neutral stationary distribution, each individual plays strategy *A* with probability one-half, i.e., ${\langle {s}_{l}\rangle }_{0}=1/2$ for all *l*. Furthermore, due to the independence of individuals’ strategy updating, ${\langle {s}_{l}{s}_{k}\rangle }_{0}={\langle {s}_{l}\rangle }_{0}{\langle {s}_{k}\rangle }_{0}=1/4$ when *l* ≠ *k*. Based on these, we have that the correlations of strategies at the neutral stationary distribution are

10 $${\langle {s}_{l}{s}_{k}\rangle }_{0}=\frac{1+{\delta }_{lk}}{4},$$

where *δ*_*lk*_ = 0 if *l* ≠ *k* and *δ*_*lk*_ = 1 if *l* = *k*. Equation (10) indicates that aspiration dynamics do not lead to assortment of strategies in the neutral stationary distribution.

### Personalized and symmetric aspirations

For personalized and symmetric aspirations, each individual *l* has its own aspiration *α*_*l*_ (*l* = 1, 2, ⋯ , *N*) and this aspiration does not depend on the strategy of individual *l*. In this case, we have

11 $$h({\bf{s}})=\frac{1}{N}\mathop{\sum }\limits_{l=1}^{N}\frac{{g}_{l}^{\prime}(0)}{{g}_{l}(0)}\left[(1-2{s}_{l}){\alpha }_{l}+(1-2{s}_{l}){\pi }_{l}({\bf{s}})\right],$$

and

12 $${\langle h({\bf{s}})\rangle }_{0}=\frac{1}{4N}\left(\mathop{\sum }\limits_{l=1}^{N}\frac{{g}_{l}^{\prime}(0)}{{g}_{l}(0)}\right)(a+b-c-d).$$

Since ${g}_{l}^{\prime}(0)\;> \; 0$ and *g*_*l*_(0) > 0 for any *l*, equation (12) implies that for symmetric aspirations, strategy *A* prevails over *B* if *a* + *b* > *c* + *d* and strategy *B* prevails over *A* if *a* + *b* < *c* + *d*. Note that the condition for strategy success does not depend on the population structure at all, which highlights the intrinsic difference between the evolutionary dynamics induced by aspiration-based and imitation-based update rules.

### Asymmetric aspirations contingent on strategies

For aspirations contingent on the strategy in use, individuals playing strategy *A* have aspiration level *α*_*A*_ while those using *B* have *α*_*B*_. Since coefficients **c** is evaluated at *β* = 0, the asymmetry of aspirations thus does not affect **c**. However, *h*(**s**) now depends on both *α*_*A*_ and *α*_*B*_, and it is modified as

13 $$h({\bf{s}})=\frac{1}{N}\mathop{\sum }\limits_{l=1}^{N}\frac{{g}_{l}^{\prime}(0)}{{g}_{l}(0)}\left[(1-{s}_{l}){\alpha }_{B}-{s}_{l}{\alpha }_{A}+(1-2{s}_{l}){\pi }_{l}({\bf{s}})\right].$$

Meanwhile, at the neutral stationary distribution, strategy correlations ${\langle {s}_{l}{s}_{k}\rangle }_{0}$ are also independent of aspirations. Based on these, we have

14 $${\langle h({\bf{s}})\rangle }_{0}=\frac{1}{4N}\left(\mathop{\sum }\limits_{l=1}^{N}\frac{{g}_{l}^{\prime}(0)}{{g}_{l}(0)}\right)(a+b-c-d+2{\alpha }_{B}-2{\alpha }_{A}),$$

which implies under asymmetric aspirations, strategy *A* prevails over *B* if

$$a+b\;> \;c+d-2({\alpha }_{B}-{\alpha }_{A}),$$

and strategy *B* prevails over *A* if *a* + *b* < *c* + *d* − 2(*α*_*B*_ − *α*_*A*_).

### Reporting summary

Further information on research design is available in the Nature Research Reporting Summary linked to this article.

## Supplementary information

Supplementary Information

Reporting Summary

## Data Availability

All data generated or analyzed during this study are included within the paper and its supplementary information files.

## Code availability

We use Microsoft Visual Studio Community 2019 and Matlab 2020a to write the custom Fortran code and analyze the data, respectively. The computer codes for Monte Carlo simulations used in this work are available in Zenodo with the identifier 10.5281/zenodo.4723407.
