# Supplementary material for: Aspiration dynamics generate robust predictions in heterogeneous populations
Source: Nat Commun. 2021 May 31;12:3250. doi: 10.1038/s41467-021-23548-4 (PMC8166829; doi:10.1038/s41467-021-23548-4)
Supplement: Supplementary file 1 — Supplementary Information [file 41467_2021_23548_MOESM1_ESM.pdf]

Supplementary Information for

Aspiration dynamics generate robust predictions in heterogeneous populations

Lei Zhou, Bin Wu, Jinming Du, and Long Wang

Contents

Supplementary Note 1: Overview 2
Supplementary Note 2: Model and notation 2
2.1 Population structure 2
2.2 Discrete-time Markov chain with finite states 2
2.3 Payoff 2
2.4 Aspiration-based update functions 2
2.5 Transitions under aspiration dynamics 3
Supplementary Note 3: Condition for strategy success 3
3.1 General condition 3
3.2 The condition for aspiration dynamics 4
3.2.1 Calculating the accumulated average abundance difference 4
3.2.2 Deriving the exact formula 5
3.2.3 Asymmetric aspirations 6
3.2.4 Accumulated payoffs 7
3.3 The condition for pairwise comparison rules 7
3.3.1 The general formula 7
3.3.2 σ for regular graphs 10
3.3.3 σ for star graphs 10
Supplementary Figure 1 Sensitivity of imitative dynamics to decision-making functions under minimal heterogeneity 11
Supplementary Figure 2 Average abundance of strategy A in different social dilemmas 12
Supplementary Figure 3 Robustness of aspiration dynamics vs. sensitivity of imitative dynamics to heterogeneity of decision-making functions on large scale-free networks . 13
Supplementary References 14

Supplementary Note 1: Overview

The supplementary text is organized as follows. First, we introduce the model and define the notations. Then we derive the general condition for strategy success, which applies to a large class of evolutionary dynamics. After that, we focus on aspiration dynamics on weighted networks and derive the exact formula of condition for strategy success under different model assumptions, for instance, symmetric or asymmetric aspirations, and average or accumulated payoffs. For a better comparison, we also prove a Proposition that connects our approach to those in [1, 2] for imitation-based update rules. Meanwhile, we give the condition for strategy success under pairwise comparison rules [3, 4].

Supplementary Note 2: Model and notation

2.1 Population structure

The population size is N and the structure of the population is depicted by an undirected weighted graph with edge weights $w_{ij} \geq 0$. This graph is fixed during the whole evolution process. Here, $w_{ij} > 0$ denotes the weight of edge ij while $w_{ij} = 0$ means there is no connection between node i and j. Since edges are undirected, $w_{ij} = w_{ji}$. Moreover, we define the weighted degree of node i as $d_i = \sum_{j=1}^{N} w_{ij}$ [1].

2.2 Discrete-time Markov chain with finite states

In our model, individuals can play one of the two strategies, strategy A and strategy B. We consider the stochastic aspiration dynamics in structured populations. The state of the system can be described by a (column) binary vector $\mathbf{s} = (s_1, s_2, \cdots, s_l, \cdots, s_N)^{\mathrm{T}}$ where $\mathbf{s}(l) = s_l$ is the strategy of individual l: $s_l = 1$ if individual l uses strategy A, $s_l = 0$ if it uses B. We model the resulting evolutionary process as a discrete-time Markov chain $\{\mathbf{S}_n, n = 0, 1, 2, 3, \cdots\}$ with state space $\{\mathbf{s}_1, \mathbf{s}_2, \cdots, \mathbf{s}_M\}$ where $M = 2^N$. For each individual l, its strategy at the k-th step is thus $\mathbf{S}_k(l)$. Note that throughout this supplementary text, all the vectors are column vectors by default.

2.3 Payoff

On the weighted graph, nodes are occupied by individuals and links indicate who interacts with whom. Individuals interact with their nearest neighbors according to the game given by the following payoff matrix

$$\begin{array}{cc} & \begin{array}{cc} A & B \end{array} \\ \begin{array}{c} A \\ B \end{array} & \begin{pmatrix} a & b \\ c & d \end{pmatrix} \end{array}, \tag{1}$$

and they collect the degree-weighted average payoff π. Here, self-interactions are excluded and each individual must have at least one neighbor to interact with, which requires that $w_{ll} = 0$ and $d_l > 0$ for all l.

For each individual l, the number of times it interacts with an A-player at state $\mathbf{s}$ is $\sum_{k=1}^{N} w_{lk} s_k$ and that with a B-player is $\sum_{k=1}^{N} w_{lk}(1 - s_k)$. The payoff of individual l at state $\mathbf{s}$ is thus

$$\pi_l(\mathbf{s}) = \sum_{k=1}^{N} \frac{w_{lk}}{d_l} \left[a s_l s_k + b s_l (1 - s_k) + c(1 - s_l) s_k + d(1 - s_l)(1 - s_k) \right]. \tag{2}$$

2.4 Aspiration-based update functions

In the real world, different individuals may make distinct decisions for the same shortfall of payoffs, which means they may have individualized ways of self-evaluation. Such heterogeneity can be characterized by

different update functions [5, 6]. We define individual l's update function as $g_l(u)$ $(l = 1, 2, \cdots, N)$ and these functions represent the tendency to switch strategies. For aspiration-based update functions, $u = \beta(\alpha_l - \pi_l)$, where α_l is individual l's aspiration level, π_l is its payoff, and $\beta > 0$ is the intensity of selection [7, 1]. Weak selection means $\beta \ll 1$ and $\beta = 0$ is the neutral drift [8]. In addition, each function $g_l(u)$ should satisfy the following restrictions:

- it is a probability, i.e., $g_l(u) \in [0, 1]$ for $u \in \mathbb{R}$;
- it is a strictly increasing function of u, i.e., $g_l'(u) = dg_l(u)/du > 0$ for all u, which indicates that individuals with higher payoffs should have a lower tendency to switch;
- $g_l(0) > 0$, which avoids frozen dynamics at the neutral drift.

2.5 Transitions under aspiration dynamics

Under aspiration dynamics, individuals' self-assessment of strategies triggers the transition between states. At each time step, only one individual can update its strategy, either switch to the other strategy or keep the current strategy unchanged. For the former, it means that the one-step transition between two distinct states of the associated Markov chain happens only if they possess the same entries everywhere except at one place which marks the strategy switching of the selected individual. Formally, this means that the one-step transition between $\mathbf{s}_i$ and $\mathbf{s}_j$ $(i \neq j)$ is possible only if $\sum_l |\mathbf{s}_i(l) - \mathbf{s}_j(l)| = 1$. Here, the operator $|\cdot|$ means to take the absolute value. For convenience, we denote the set of states which can transit out of (or into) $\mathbf{s}_i$ as $\mathcal{N}_i$ $(\mathbf{s}_i \notin \mathcal{N}_i)$, namely, the neighboring states of $\mathbf{s}_i$. If the selected individual instead does not switch, the state of the Markov chain stays unchanged. Therefore, the transition probabilities of the Markov chain are

$$p_{ij} = \begin{cases} \frac{1}{N} g_{l_{ij}}(\beta(\alpha_{l_{ij}} - \pi_{l_{ij}}(\mathbf{s}_i))), & \text{if } j \in \mathcal{N}_i, \\ 1 - \sum_{k \in \mathcal{N}_i} \frac{1}{N} g_{l_{ik}}(\beta(\alpha_{l_{ik}} - \pi_{l_{ik}}(\mathbf{s}_i))), & \text{if } i = j, \\ 0, & \text{otherwise,} \end{cases} \tag{3}$$

where l_{ij} (l_{ik}) is the selected individual and it is uniquely determined by $\mathbf{s}_i$ and $\mathbf{s}_j$ $(\mathbf{s}_k)$. A simple way to locate l_{ij} is by looking at the (unique) non-zero entry of vector $\mathbf{s}_j - \mathbf{s}_i$ when $\mathbf{s}_j \in \mathcal{N}_i$. With all these transition probabilities, we have a transition matrix $\mathbf{P}$ whose (i, j)-th entry is p_{ij}, which describes the one-step transition probability between any states.

Supplementary Note 3: Condition for strategy success

3.1 General condition

Let us denote the number of individuals playing strategy A at state $\mathbf{s}$ as $\phi(\mathbf{s}) = \sum_{l=1}^{N} s_l$. The abundance (frequency) of strategy A and B at state $\mathbf{s}$ is $x_A = \phi(\mathbf{s})/N$ and $x_B = 1 - x_A$, respectively. The frequencies of strategy A at all states are denoted as a vector $\mathbf{x} = (\frac{\phi(\mathbf{s}_1)}{N}, \frac{\phi(\mathbf{s}_2)}{N}, \cdots, \frac{\phi(\mathbf{s}_{M-1})}{N}, \frac{\phi(\mathbf{s}_M)}{N})^{\mathrm{T}}$ and those of B are $\mathbf{1} - \mathbf{x}$ where $\mathbf{1}$ is a vector with all its elements being one. Under aspiration dynamics, transition is possible between all the states (not necessarily in one step) and the number of steps taken can be odd or even. This indicates that aspiration dynamics admit a unique stationary distribution $\mathbf{u}$, in which each state occurs with a positive probability [9] and all these probabilities sum up to one, i.e., $\mathbf{u}^{\mathrm{T}}\mathbf{1} = 1$. The stationary distribution $\mathbf{u}$ can be obtained by solving a set of linear equations $\mathbf{u}^{\mathrm{T}}\mathbf{P} = \mathbf{u}^{\mathrm{T}}$. In this stationary distribution, we calculate the average abundance of strategy A as $\langle x_A \rangle = \mathbf{u}^{\mathrm{T}}\mathbf{x}$ and that of B as $\langle x_B \rangle = \mathbf{u}^{\mathrm{T}}(\mathbf{1} - \mathbf{x}) = 1 - \langle x_A \rangle$, where the angle bracket $\langle \cdot \rangle$ means to take the average over all the states.

Strategy A is more abundant than strategy B if and only if $\langle x_A \rangle > \frac{1}{2}$ [8, 10]. Since $\langle x_A \rangle + \langle x_B \rangle = \langle x_A + x_B \rangle = 1$, the condition $\langle x_A \rangle > \frac{1}{2}$ is equivalent to $\langle x_A \rangle > \langle x_B \rangle$ [11, 12], and further equivalent to

$\langle x_A - x_B \rangle = \mathbf{u}^{\mathrm{T}}(2\mathbf{x} - \mathbf{1}) > 0$. Note that $\mathbf{u}$ and $\mathbf{P}$ depend on the selection intensity β and so do $\langle x_A \rangle$ and $\langle x_B \rangle$. To show this dependence, we rewrite them as $\mathbf{u}_\beta$, $\mathbf{P}_\beta$, $\langle x_A \rangle_\beta$, and $\langle x_B \rangle_\beta$. In the limit of weak selection $\beta \to 0$, we do a Talyor expansion of $\langle x_A - x_B \rangle_\beta$ with respect to β at $\beta = 0$ and get

$$\langle x_A - x_B \rangle_\beta = \langle x_A - x_B \rangle_0 + \langle x_A - x_B \rangle_0' \beta + O(\beta^2), \tag{4}$$

where $\langle x_A - x_B \rangle_0'$ is the first-order derivative of $\langle x_A - x_B \rangle_\beta$ with respect to β, evaluated at $\beta = 0$ and $O(\beta^2)$ is the higher order terms of β^2, which is negligible as $\beta \to 0$. Note that the first term in the right-hand side of (4) is the average abundance difference between strategy A and B at the neutral drift, which is zero [5]. Since $\beta > 0$, the condition for strategy A to be more abundant than B becomes

$$\langle x_A - x_B \rangle_0' = (\mathbf{u}_0')^{\mathrm{T}}(2\mathbf{x} - \mathbf{1}) > 0. \tag{5}$$

To get $\mathbf{u}_0'$, we differentiate $\mathbf{u}_\beta^{\mathrm{T}} \mathbf{P}_\beta = \mathbf{u}_\beta^{\mathrm{T}}$ with respect to β and evaluate it at $\beta = 0$, by rearranging items, we obtain

$$(\mathbf{u}_0')^{\mathrm{T}}(\mathbf{I} - \mathbf{P}_0) = \mathbf{u}_0^{\mathrm{T}} \mathbf{P}_0', \tag{6}$$

where $\mathbf{I}$ is the identity matrix of dimension M, $\mathbf{u}_0' = \frac{d}{d\beta}\mathbf{u}_\beta|_{\beta=0}$, and $\mathbf{P}_0' = \frac{d}{d\beta}\mathbf{P}_\beta|_{\beta=0}$. Note that $\mathbf{I} - \mathbf{P}_0$ is not invertible since it is not full rank. Introduce the matrix $\mathbf{W} = \mathbf{1}\mathbf{u}_0^{\mathrm{T}}$, we know that the matrix $\mathbf{I} - \mathbf{P}_0 + \mathbf{W}$ is invertible and its inverse, denoted as $\mathbf{Z}_0$, is called the *fundamental matrix* [9]. As shown in ref. [9], $\mathbf{Z}_0 = (\mathbf{I} - \mathbf{P}_0 + \mathbf{W})^{-1} = \sum_{k=0}^{\infty}(\mathbf{P}_0 - \mathbf{W})^k = \mathbf{I} + \sum_{k=1}^{\infty}(\mathbf{P}_0^k - \mathbf{W})$ and $(\mathbf{I} - \mathbf{P}_0)\mathbf{Z}_0 = \mathbf{I} - \mathbf{W}$. Note that $(\mathbf{u}_0')^{\mathrm{T}}\mathbf{W} = \mathbf{0}^{\mathrm{T}}$, right multiply $\mathbf{Z}_0$ at both sides of equation (6), we have

$$(\mathbf{u}_0')^{\mathrm{T}} = \mathbf{u}_0^{\mathrm{T}} \mathbf{P}_0' \mathbf{Z}_0. \tag{7}$$

Substituting equation (7) into (5), we have that strategy A is favored over strategy B if

$$\langle x_A - x_B \rangle_0' = \mathbf{u}_0^{\mathrm{T}} \mathbf{P}_0' \mathbf{Z}_0 (2\mathbf{x} - \mathbf{1}) = \mathbf{u}_0^{\mathrm{T}} \mathbf{P}_0' \left(\mathbf{I} + \sum_{k=1}^{\infty} (\mathbf{P}_0^k - \mathbf{W}) \right) (2\mathbf{x} - \mathbf{1}) = \mathbf{u}_0^{\mathrm{T}} \mathbf{P}_0' \mathbf{c} > 0, \tag{8}$$

where $\mathbf{c} = \sum_{k=0}^{\infty} \mathbf{P}_0^k (2\mathbf{x} - \mathbf{1}) = (c_1, c_2, \cdots, c_i, \cdots, c_M)^{\mathrm{T}}$. In detail, $c_i = \sum_{k=0}^{\infty} \sum_{j=1}^{M} p_{ij}^{(k)} (\frac{2\phi(\mathbf{s}_j)}{N} - 1)$ where $p_{ij}^{(k)}$ is the (i, j)-th entry of $\mathbf{P}_0^k$. Remind that $p_{ij}^{(k)}$ is the k-step transition probability from state $\mathbf{s}_i$ to $\mathbf{s}_j$. Summing over all the possible states, $\sum_{j=1}^{M} p_{ij}^{(k)} (\frac{2\phi(\mathbf{s}_j)}{N} - 1)$ is the average abundance difference between strategy A and B at the k-th step when starting at state $\mathbf{s}_i$. Therefore, c_i is the accumulated average abundance difference during the whole evolution when the initial state is $\mathbf{s}_i$.

Actually, equation (8) holds for any evolutionary dynamics which have a unique limiting stationary distribution and an equal abundance of strategy A and B at the neutral drift. For example, the death-birth, birth-death process and pairwise comparison with symmetric mutations are of this class [13, 4, 8, 12, 14, 1].

3.2 The condition for aspiration dynamics

3.2.1 Calculating the accumulated average abundance difference

Remind that $c_i = \sum_{k=0}^{\infty} \sum_{j=1}^{M} p_{ij}^{(k)} (\frac{2\phi(\mathbf{s}_j)}{N} - 1)$ where $p_{ij}^{(k)}$ is the k-step transition probability from state i to j at the neutral drift (i.e., $\beta = 0$). It represents the accumulated average abundance difference during the whole evolution when starting at state $\mathbf{s}_i$. Here, the abundance difference at each state aggregates contributions from all the individuals. For instance, at state $\mathbf{s}_j$, individual l's contribution to the abundance difference is $\frac{2\mathbf{s}_j(l)}{N} - \frac{1}{N}$. From the perspective of individual contributions, we rewrite c_i as $\sum_{l=1}^{N} \sum_{k=0}^{\infty} \sum_{j=1}^{M} p_{ij}^{(k)} (\frac{2\mathbf{s}_j(l)}{N} - \frac{1}{N})$. The idea is that to calculate the accumulated average abundance difference during the whole evolution, we can first calculate the individual contribution and then aggregate them. This approach is made possible due to the following properties of aspiration dynamics.

Under aspiration dynamics, when $\beta = 0$, the payoffs collected from the games do not affect individuals' strategy revisions. More importantly, during the whole evolution, individuals' strategy revisions are completely independent of each other. Due to this independence, we can study the much simpler two-state Markov chains for each individual instead of dealing with the whole chain with 2^N states. For the two-state Markov chain of individual l, the state space is $\{0,1\}$ and the associated transition matrix is

$$\begin{array}{c} \\ 1 \\ 0 \end{array}\begin{array}{c} \begin{array}{cc} \quad 1 \quad & \quad 0 \quad \end{array} \\ \begin{pmatrix} 1-\frac{1}{N}g_l(0) & \frac{1}{N}g_l(0) \\ \frac{1}{N}g_l(0) & 1-\frac{1}{N}g_l(0) \end{pmatrix} \end{array}. \tag{9}$$

The associated k-step transition probabilities are

$$\begin{aligned} \text{Prob}\,(\mathbf{S}_k(l)=0|\mathbf{S}_0(l)=0) &= \text{Prob}(\mathbf{S}_k(l)=1|\mathbf{S}_0(l)=1) = \frac{1}{2}\left[1+(1-\frac{2}{N}g_l(0))^k\right], \\ \text{Prob}(\mathbf{S}_k(l)=1|\mathbf{S}_0(l)=0) &= \text{Prob}(\mathbf{S}_k(l)=0|\mathbf{S}_0(l)=1) = \frac{1}{2}\left[1-(1-\frac{2}{N}g_l(0))^k\right]. \end{aligned}$$

Since each individual's behavior is highly independent of each other, we can calculate their contribution to the accumulated average abundance difference separately. Starting at state $\mathbf{s}_i$, at step k, the contribution of individual l to the average abundance difference is

$$\begin{aligned} &\left(\frac{2\mathbf{s}_i(l)}{N}-\frac{1}{N}\right)\text{Prob}(\mathbf{S}_k(l)=\mathbf{s}_i(l)|\mathbf{S}_0(l)=\mathbf{s}_i(l)) + \left(\frac{1}{N}-\frac{2\mathbf{s}_i(l)}{N}\right)\text{Prob}(\mathbf{S}_k(l)=1-\mathbf{s}_i(l)|\mathbf{S}_0(l)=\mathbf{s}_i(l)) \\ &= \frac{2\mathbf{s}_i(l)-1}{2N}\left[1+(1-\frac{2}{N}g_l(0))^k\right] + \frac{1-2\mathbf{s}_i(l)}{2N}\left[1-(1-\frac{2}{N}g_l(0))^k\right] = \frac{2\mathbf{s}_i(l)-1}{N}\left(1-\frac{2}{N}g_l(0)\right)^k. \end{aligned}$$

By definition of the update functions, $0 < g_l(0) < 1$ for all l. The contribution of individual l to the accumulated average abundance difference during the whole evolution is thus

$$\sum_{k=0}^{\infty}\frac{2\mathbf{s}_i(l)-1}{N}\left(1-\frac{2}{N}g_l(0)\right)^k = \frac{2\mathbf{s}_i(l)-1}{N}\frac{N}{2g_l(0)} = \frac{2\mathbf{s}_i(l)-1}{2g_l(0)}.$$

Summing up all the individual contributions, we obtain the accumulated average abundance difference of the whole Markov chain as

$$c_i = \sum_{l=1}^{N}\frac{2\mathbf{s}_i(l)-1}{2g_l(0)} \tag{10}$$

By this formula, we obtain that $\mathbf{c}$ only depends on the update functions. It does not depend on the game, the aspiration levels, and the population structure.

3.2.2 Deriving the exact formula

Denote $h(\mathbf{s}_i) = \sum_{j=1}^{M} p'_{ij}c_j$, which is the i-th row of $\mathbf{P}'_0\mathbf{c}$. Equation (8) implies that the condition for strategy A to be favored over B is

$$\langle x_A - x_B\rangle'_0 = \langle h(\mathbf{s})\rangle_0 > 0, \tag{11}$$

where the bracket $\langle\cdot\rangle_0$ means to take the average over the neural stationary distribution (i.e., when $\beta = 0$).

Substitute formula (10) of c_j into $h(\mathbf{s}_i)$, we have

$$h(\mathbf{s}_i) = \sum_{j=1}^{M} p'_{ij}c_j = \sum_{j\neq i}^{M} p'_{ij}(c_j - c_i) = \sum_{j\neq i}^{M} p'_{ij}\sum_{m=1}^{N}\frac{\mathbf{s}_j(m)-\mathbf{s}_i(m)}{g_m(0)}. \tag{12}$$

Note that the transitions can happen if $\mathbf{s}_i$ and $\mathbf{s}_j$ $(i \neq j)$ differ at only one individual's strategy. Combining with the transition probability (3), we have that for $j \neq i$,

$$p'_{ij} \sum_{m=1}^{N} \frac{\mathbf{s}_j(m) - \mathbf{s}_i(m)}{g_m(0)} = \begin{cases} \frac{1}{N} \frac{g'_{l_{ij}}(0)}{g_{l_{ij}}(0)} (\alpha_{l_{ij}} - \pi_{l_{ij}}(\mathbf{s}_i)) (1 - 2\mathbf{s}_i(l_{ij})), & \text{if } j \in \mathcal{N}_i, \\ 0, & \text{otherwise.} \end{cases}$$

where $g'_{l_{ij}}(u) = dg_{l_{ij}}(u)/du$ (see Supplementary Note 2.4). The summation over all neighboring states can also be rewritten as the summation over all the individuals, which leads to

$$h(\mathbf{s}_i) = \frac{1}{N} \sum_{l=1}^{N} \frac{g'_l(0)}{g_l(0)} (\alpha_l - \pi_l(\mathbf{s}_i)) (1 - 2\mathbf{s}_i(l)).$$

Here, we drop the subscript l_{ij} since the summation goes over all the individuals.

Substituting the payoff formula (2) into the above equation and using $s_l^2 = s_l$, we obtain that for any state $\mathbf{s}$,

$$h(\mathbf{s}) = \frac{1}{N} \sum_{l=1}^{N} \frac{g'_l(0)}{g_l(0)} \left\{ (1 - 2s_l)\alpha_l + \sum_{k=1}^{N} \frac{w_{lk}}{d_l} \left[a s_l s_k + b s_l (1 - s_k) - c(1 - s_l) s_k - d(1 - s_l)(1 - s_k) \right] \right\}. \tag{13}$$

Evaluating $\langle h(\mathbf{s}) \rangle_0$ needs to analyze the correlation of strategies in the neutral stationary distribution. Under aspiration dynamics, when the selection intensity $\beta = 0$ (neutral drift), individuals' strategy updating does not dependent on the aspiration level, the payoff, and the population structure. The transition probabilities between any two states are thus the same in both directions, which indicates that the transition matrix is symmetric, i.e., $\mathbf{P}_0 = \mathbf{P}_0^{\mathrm{T}}$. By the uniqueness of the stationary distribution $\mathbf{u}_0$ and the property of the transition (stochastic) matrix $\mathbf{P}_0 \mathbf{1} = \mathbf{1}$, we have $\mathbf{1}^{\mathrm{T}} \mathbf{P}_0 = \mathbf{1}^{\mathrm{T}}$, which leads to $\mathbf{u}_0 = 2^{-N} \mathbf{1}$ through normalization. This results in that at the neutral stationary distribution, each individual plays strategy A with probability one-half, i.e., $\langle s_l \rangle_0 = 1/2$ for all l. Moreover, since individuals' strategy updating is independent of each other, $\langle s_l s_k \rangle_0 = \langle s_l \rangle_0 \langle s_k \rangle_0 = 1/4$ when $l \neq k$. Based on these, we have that the correlations of strategies at the neutral stationary distribution are

$$\langle s_l s_k \rangle_0 = \frac{1 + \delta_{lk}}{4}, \tag{14}$$

where $\delta_{lk} = 0$ if $l \neq k$ and $\delta_{lk} = 1$ if $l = k$. Equation (14) indicates that aspiration dynamics do not lead to assortment of strategies in the neutral stationary distribution.

Combining equations (11), (14), and (13), we obtain that

$$\langle x_A - x_B \rangle'_0 = \frac{1}{4N} \left(\sum_{l=1}^{N} \frac{g'_l(0)}{g_l(0)} \right) (a + b - c - d). \tag{15}$$

Since $g_l(0) > 0$, $g'_l(0) > 0$ by definition, strategy A outcompetes B in abundance if $a + b > c + d$ and strategy B outcompete A if $a + b < c + d$.

Moreover, the average abundance of strategy A in the stationary distribution can be obtained by combining equation (15) and the identity $\langle x_A + x_B \rangle_\beta = 1$, which leads to

$$\langle x_A \rangle_\beta = \frac{1}{2} + \frac{1}{8N} \left(\sum_{l=1}^{N} \frac{g'_l(0)}{g_l(0)} \right) (a + b - c - d)\beta + O(\beta^2). \tag{16}$$

3.2.3 Asymmetric aspirations

For aspirations contingent on the strategy in use, individuals playing strategy A have aspiration level α_A while those using B have α_B. As shown in Supplementary Note 3.2.1, coefficients $\mathbf{c}$ in equation (8) is evaluated

at $\beta = 0$. The asymmetry of aspirations thus does not affect $\mathbf{c}$. However, $h(\mathbf{s})$ in Supplementary Note 3.2.2 now depends on both α_A and α_B, and equation (13) is modified as

$$h(\mathbf{s}) = \frac{1}{N}\sum_{l=1}^{N}\frac{g_l'(0)}{g_l(0)}\left\{(1-s_l)\alpha_B - s_l\alpha_A + \sum_{k=1}^{N}\frac{w_{lk}}{d_l}\left[as_ls_k + bs_l(1-s_k) - c(1-s_l)s_k - d(1-s_l)(1-s_k)\right]\right\}. \tag{17}$$

Note that at the neutral stationary distribution, strategy correlations $\langle s_l s_k\rangle_0$ are independent of aspirations. Combining equations (11), (14), and (17), we obtain that

$$\langle x_A - x_B\rangle_0' = \frac{1}{4N}\left(\sum_{l=1}^{N}\frac{g_l'(0)}{g_l(0)}\right)(a+b-c-d+2\alpha_B-2\alpha_A). \tag{18}$$

Therefore, under the limit of weak selection, the condition for strategy A to prevail over B for asymmetric aspirations is

$$a+b > c+d-2(\alpha_B-\alpha_A),$$

and that for B to prevail A is $a+b < c+d-2(\alpha_B-\alpha_A)$.

In the Prisoner's Dilemma game (i.e., $a<c$ and $b<d$), $a+b$ is always less than $c+d$. If the aspirations are symmetric, strategy A is never favored since $a+b<c+d$. With asymmetric aspirations, if $\alpha_B-\alpha_A$ is large enough, it is possible that $a+b>c+d-2(\alpha_B-\alpha_A)$, which selects strategy A over B. Considering the evolution of cooperation, this means that cooperation can prevail over defection if individuals aspire more when they defect.

3.2.4 Accumulated payoffs

Under accumulated payoffs, the payoff of individual l at state $\mathbf{s}$ is

$$\pi_l(\mathbf{s}) = \sum_{k=1}^{N} w_{lk}\left[as_ls_k + bs_l(1-s_k) + c(1-s_l)s_k + d(1-s_l)(1-s_k)\right]. \tag{19}$$

This leads to that

$$h(\mathbf{s}) = \frac{1}{N}\sum_{l=1}^{N}\frac{g_l'(0)}{g_l(0)}\left\{(1-2s_l)\alpha_l + \sum_{k=1}^{N} w_{lk}\left[as_ls_k + bs_l(1-s_k) - c(1-s_l)s_k - d(1-s_l)(1-s_k)\right]\right\}.$$

Since the way of payoff collection does not affect the correlation of strategies in the neutral stationary distribution, we obtain that

$$\langle x_A - x_B\rangle_0' = \frac{1}{4N}\left(\sum_{l=1}^{N}d_l\frac{g_l'(0)}{g_l(0)}\right)(a+b-c-d) \tag{20}$$

and the condition for strategy A to be favored over B is $a+b>c+d$ and that for strategy B to be favored over A is $a+b<c+d$. This means that accumulated payoffs do not change the condition for strategy success under aspiration dynamics.

3.3 The condition for pairwise comparison rules

3.3.1 The general formula

Pairwise comparison rule is an imitation-based update rule [3, 4]. At each time step, a focal individual l is randomly selected. Then it randomly selects a role model m from its neighbors proportional to weights and copy the strategy of individual m based on the payoff difference $\Delta\pi$. When mutation or random exploration is considered, individual l will use this update rule with probability $1-\mu$ ($0<\mu<1$) or switch to a

random strategy with probability μ. Here, we assume that all the individuals in the population use the same imitation-based update function $g(u)$ where $u = \beta\Delta\pi$. Note that such update function satisfies the same restrictions as mentioned in Supplementary Note 2.4. For pairwise comparison rule, the probability for individual l to adopt the strategy of individual m is $(1-\mu)g(\beta\Delta\pi) + \mu/2$ where $\Delta\pi = \pi_m - \pi_l$.

Define the degree-weighted frequency of strategy A in state $\mathbf{s}$ as $\hat{x}_A(\mathbf{s}) = \sum_{l=1}^{N} d_l s_l/\Omega$ where $\Omega = \sum_{l=1}^{N} d_i$ [1]. In the neutral stationary distribution, the average degree-weighted abundance of strategy A equals to that of B, i.e., $\langle \hat{x}_A \rangle_0 = \langle \hat{x}_B \rangle_0 = \frac{1}{2}$. Under the limit of weak selection $\beta \to 0$, using equation (7), we have

$$\langle \hat{x}_A - \hat{x}_B \rangle_0' = \mathbf{u}_0^{\mathrm{T}} \mathbf{P}_0' \mathbf{Z}_0 (2\hat{\mathbf{x}} - \mathbf{1}). \tag{21}$$

In the following, we will try to prove a Proposition which connects our approach to those in [1, 2].

Proposition 1. *With mutation rate $0 < \mu < 1$, under the neutral drift $\beta = 0$, $2\hat{\mathbf{x}} - \mathbf{1}$ is an eigenvector of $\mathbf{Z}_0$ with respect to the eigenvalue $\frac{N}{\mu}$.*

Proof. By definition, $\mathbf{Z}_0 = (\mathbf{I} - \mathbf{P}_0 + \mathbf{W})^{-1}$. If $2\hat{\mathbf{x}} - \mathbf{1}$ is an eigenvector of $\mathbf{I} - \mathbf{P}_0 + \mathbf{W}$ with respect to the eigenvalue μ/N, then Proposition 1 is proved.

Here, we first calculate $\mathbf{P}_0(2\hat{\mathbf{x}} - \mathbf{1})$. The ith-row of $\mathbf{P}_0(2\hat{\mathbf{x}} - \mathbf{1})$ is

$$\sum_{j=1}^{M} p_{ij}^{\circ}(2\hat{x}_A(\mathbf{s}_j) - 1) = 2\sum_{j=1}^{M} p_{ij}^{\circ}\hat{x}_A(\mathbf{s}_j) - 1 = 2\sum_{j=1}^{M} p_{ij}^{\circ}\left(\hat{x}_A(\mathbf{s}_j) - \hat{x}_A(\mathbf{s}_i)\right) + 2\hat{x}_A(\mathbf{s}_i) - 1, \tag{22}$$

where the subscript $^{\circ}$ represents the neutral drift ($\beta = 0$). The first term on the right hand of equation (22) is the expected instantaneous rate of degree-weighted frequency change [1], which equals to zero when mutation is absent. With mutations, $\sum_{j=1}^{M} p_{ij}^{\circ}\left(\hat{x}_A(\mathbf{s}_j) - \hat{x}_A(\mathbf{s}_i)\right)$ is non-zero due to the contribution of mutations. We thus have

$$\sum_{j=1}^{M} p_{ij}^{\circ}\left(\hat{x}_A(\mathbf{s}_j) - \hat{x}_A(\mathbf{s}_i)\right) = \frac{1}{N}\sum_{l=1}^{N}\left[\mathbf{s}_i(l)\frac{\mu}{2}\left(-\frac{d_l}{\Omega}\right) + (1 - \mathbf{s}_i(l))\frac{\mu}{2}\frac{d_l}{\Omega}\right] = \frac{\mu}{2N}(1 - 2\hat{x}_A(\mathbf{s}_i)) \tag{23}$$

Combining equations (22) and (23), we get

$$\mathbf{P}_0(2\hat{\mathbf{x}} - \mathbf{1}) = (1 - \frac{\mu}{N})(2\hat{\mathbf{x}} - \mathbf{1}). \tag{24}$$

This leads to

$$(\mathbf{I} - \mathbf{P}_0 + \mathbf{W})(2\hat{\mathbf{x}} - \mathbf{1}) = 2\hat{\mathbf{x}} - \mathbf{1} - (1 - \frac{\mu}{N})(2\hat{\mathbf{x}} - \mathbf{1}) = \frac{\mu}{N}(2\hat{\mathbf{x}} - \mathbf{1}), \tag{25}$$

which proves Proposition 1.

By Proposition 1, equation (21) becomes

$$\langle \hat{x}_A - \hat{x}_B \rangle_0' = \frac{N}{\mu}\mathbf{u}_0^{\mathrm{T}}\mathbf{P}_0'(2\hat{\mathbf{x}} - \mathbf{1}) = \frac{2N}{\mu}\mathbf{u}_0^{\mathrm{T}}\mathbf{P}_0'\hat{\mathbf{x}}. \tag{26}$$

Note that the ith-row of $\mathbf{P}_0'\hat{\mathbf{x}}$ is

$$\sum_{j=1}^{M} p_{ij}'\hat{x}_A(\mathbf{s}_j) = \sum_{j=1}^{M} p_{ij}'(\hat{x}_A(\mathbf{s}_j) - \hat{x}_A(\mathbf{s}_i)) = (1 - \mu)\hat{\Delta}_{\mathrm{sel}}'(\mathbf{s}_i) \tag{27}$$

where $\hat{\Delta}_{\mathrm{sel}}'(\mathbf{s}_i)$ is the first-order coefficient of the reproductive value weighted change due to selection [1, 2]. Now equation (26) can be rewritten as

$$\langle \hat{x}_A - \hat{x}_B \rangle_0' = \frac{N}{\mu}\mathbf{u}_0^{\mathrm{T}}\mathbf{P}_0'(2\hat{\mathbf{x}} - \mathbf{1}) = \frac{2N(1 - \mu)}{\mu}\langle \hat{\Delta}_{\mathrm{sel}}' \rangle_0. \tag{28}$$

Therefore, $\langle \hat{x}_A \rangle > \langle \hat{x}_B \rangle$ if and only if $\langle \hat{\Delta}_{\mathrm{sel}}' \rangle_0 > 0$. Up to now, we have demonstrated that our approach is equivalent to that in [1, 2] for homogeneous imitation-based update rules. In what follows, we will use

their results to derive the condition for strategy success for pairwise comparison rules under the limit of rare mutation ($\mu \to 0$) and weak selection ($\beta \to 0$).

By Corollary 1 in [2], under rare mutation $\mu \to 0$, $\langle x_A \rangle > \langle x_B \rangle$ is equivalent to $\langle \hat{x}_A \rangle > \langle \hat{x}_B \rangle$, which is also equivalent to $\frac{d}{d\mu}\langle \hat{\Delta}'_{\text{sel}} \rangle_0|_{\mu=0} > 0$. Here, for the pairwise comparison rule,

$$\hat{\Delta}'_{\text{sel}}(\mathbf{s}) = \sum_{l=1}^{N}\sum_{k=1}^{N} \frac{w_{lk}}{\Omega} g'(0)(s_k - s_l)(\pi_k(\mathbf{s}) - \pi_l(\mathbf{s})). \tag{29}$$

Inserting equation (2) into equation (29) and for specific payoff values $a = \mathcal{B} - \mathcal{C}, b = -\mathcal{C}, c = \mathcal{B}, d = 0$ where $\mathcal{C} > 0$, we have

$$\begin{aligned}
\langle \hat{\Delta}'_{\text{sel}} \rangle_0 &= \sum_{l=1}^{N}\sum_{k=1}^{N} \frac{w_{lk}}{\Omega} g'(0)\langle (s_k - s_l)(\pi_k(\mathbf{s}) - \pi_l(\mathbf{s}))\rangle_0 \\
&= \sum_{l=1}^{N}\sum_{k=1}^{N} \frac{w_{lk}}{\Omega} g'(0) \left[\mathcal{B} \sum_{m=1}^{N} \frac{w_{km}}{d_k} \left(\langle x_m x_k \rangle_0 - \langle x_m x_l \rangle_0\right) \right. \\
&\quad \left. - \mathcal{B} \sum_{m=1}^{N} \frac{w_{lm}}{d_l} \left(\langle x_m x_k \rangle_0 - \langle x_m x_l \rangle_0\right) - \mathcal{C}\left(\langle x_k \rangle_0 - 2\langle x_k x_l \rangle_0 + \langle x_l \rangle_0\right) \right].
\end{aligned} \tag{30}$$

At the neutral stationary distribution, evaluating the assortment of types $\langle x_i x_j \rangle_0$ needs to know the identity-by-descend probabilities q_{ij}. Following [1], their relation reads $\langle x_i x_j \rangle_0 = (1 + q_{ij})/4$. As a special case, when $i = j$, $\langle x_i^2 \rangle_0 = \langle x_i \rangle_0 = 1/2$ since $q_{ii} = 1$ for all i. Furthermore, under rare mutation $\mu \to 0$, the identity-by-descend probabilities q_{ij} has the low-mutation expansion $q_{ij} = 1 - \mu\tau_{ij} + O(\mu^2)$ [15, 1], which connects $\langle x_i x_j \rangle_0$ to coalescence times τ_{ij} by

$$\langle x_i x_j \rangle_0 = \frac{1}{2} - \frac{1}{4}\tau_{ij}\mu + O(\mu^2). \tag{31}$$

To calculate τ_{ij}, we first introduce the quantity $\tilde{\tau}_{ij} = g(0)\tau_{ij}$. Analogous to other imitation-based update rules, we have that for pairwise comparison rules, $\tilde{\tau}_{ij}$ satisfy the following recurrent relations:

$$\tilde{\tau}_{ml} = \begin{cases} 1 + \frac{1}{2}\sum_{k=1}^{N}\left(\frac{w_{mk}}{d_m}\tilde{\tau}_{kl} + \frac{w_{lk}}{d_l}\tilde{\tau}_{mk}\right), & m \neq l; \\ 0, & m = l. \end{cases} \tag{32}$$

Note that the form of the above relations is exactly the same as those for the death-birth update rule [1]. Therefore, at the neutral drift, the rescaled coalescence times $\tilde{\tau}_{ij}$ under the pairwise comparison rule equals to the coalescence times τ_{ij} under the death-birth update rule.

Inserting the relation (31) into equation (30), we get

$$\begin{aligned}
\langle \hat{\Delta}'_{\text{sel}} \rangle_0 &= \frac{\mu}{4} g'(0) \left[(-2\mathcal{C}) \sum_{l=1}^{N}\sum_{k=1}^{N} \frac{w_{lk}}{\Omega}\tau_{lk} + \mathcal{B}\sum_{k=1}^{N}\sum_{l=1}^{N}\sum_{m=1}^{N} \frac{w_{lk}w_{km}}{d_k\Omega}(\tau_{ml} - \tau_{mk}) \right. \\
&\quad \left. - \mathcal{B}\sum_{k=1}^{N}\sum_{l=1}^{N}\sum_{m=1}^{N} \frac{w_{lk}w_{lm}}{d_l\Omega}(\tau_{ml} - \tau_{mk}) \right] + O(\mu^2).
\end{aligned} \tag{33}$$

Since $g'(0) > 0$, using equation (33) we have that $\frac{d}{d\mu}\langle \hat{\Delta}'_{\text{sel}} \rangle_0|_{\mu=0} > 0$ is equivalent to

$$(-2\mathcal{C}) \sum_{l=1}^{N}\sum_{k=1}^{N} \frac{w_{lk}}{\Omega}\tau_{lk} + \mathcal{B}\sum_{k=1}^{N}\sum_{l=1}^{N}\sum_{m=1}^{N} \frac{w_{lk}w_{km}}{d_k\Omega}(\tau_{ml} - \tau_{mk}) - \mathcal{B}\sum_{k=1}^{N}\sum_{l=1}^{N}\sum_{m=1}^{N} \frac{w_{lk}w_{lm}}{d_l\Omega}(\tau_{ml} - \tau_{mk}) > 0. \tag{34}$$

To simplify the above inequality, we define the probability to step from node i to node j in a one-step random walk as $v_{ij} = w_{ij}/d_i$ and the probability for an n-step random walk as $v_{ij}^{(n)} = \sum_{m=1}^{N} v_{im}^{(n-1)} v_{mj}$.

By introducing the quantity $\tilde{\tau}^{(n)} = \sum_{k=1}^{N}\sum_{l=1}^{N}\frac{d_k}{\Omega}v_{kl}^{(n)}\tilde{\tau}_{kl} = g(0)\sum_{k=1}^{N}\sum_{l=1}^{N}\frac{d_k}{\Omega}v_{kl}^{(n)}\tau_{kl}$ and multiplying both sides of inequality (34) by $g(0)$, we have that $\langle x_A\rangle > \langle x_B\rangle$ is equivalent to

$$-2\mathcal{C}\tilde{\tau}^{(1)} + 2\mathcal{B}\left(\tilde{\tau}^{(2)} - \tilde{\tau}^{(1)}\right) > 0. \tag{35}$$

For graphs without self-loops, $\tilde{\tau}^{(2)} - \tilde{\tau}^{(1)} = -1$ [1]. Therefore, under rare mutation and weak selection, strategy A outcompetes B in abundance (i.e., $\langle x_A\rangle > \langle x_B\rangle$) if and only if

$$\frac{\mathcal{B}}{\mathcal{C}} < -\tilde{\tau}^{(1)}. \tag{36}$$

Note that $\tilde{\tau}^{(1)} = \frac{g(0)}{\Omega}\sum_{k=1}^{N}\sum_{l=1}^{N} w_{kl}\tau_{kl} > 0$. This indicates that under pairwise comparison rules, cooperation can never evolve on any weighted network. The reason is that for strategy A to be a cooperative behavior, the benefit $\mathcal{B}$ must exceed the cost $\mathcal{C}$ (i.e., $\mathcal{B} > \mathcal{C}$). However, if $\mathcal{B} > \mathcal{C}$, cooperation is never favored by natural selection according to condition (36).

In addition, condition (36) implies that the critical benefit-to-cost ratio $(\mathcal{B}/\mathcal{C})^*$ is $-\tilde{\tau}^{(1)}$. Using the Structure Coefficient Theorem [8], for a general game with payoff values a, b, c, d, $\langle x_A\rangle > \langle x_B\rangle$ if and only if $\sigma a + b > c + \sigma d$ where $\sigma = ((\mathcal{B}/\mathcal{C})^* + 1)\,/\,((\mathcal{B}/\mathcal{C})^* - 1)$. Here, we get that for the pairwise comparison rule,

$$\sigma = \frac{\tilde{\tau}^{(1)} - 1}{\tilde{\tau}^{(1)} + 1}. \tag{37}$$

3.3.2 σ for regular graphs

To calculate σ for regular graphs, we use the results from Section 5.3 of the supplementary information for [1]. The results imply that $\tilde{\tau}^{(1)} = N - 1$. Substituting this into equation (37), we have that under under pairwise comparison rules,

$$\sigma = \frac{N-2}{N} \tag{38}$$

for regular graphs.

3.3.3 σ for star graphs

For star graphs, the node in the center is called the hub (H) and those connecting to the hub are called the leaves (L). Due to the symmetry, we have that the rescaled coalescence times between any two (different) leaves are the same, which is denoted as $\tilde{\tau}_{LL}$. Similarly, the rescaled coalescence times between the hub and a leaf is denoted as $\tilde{\tau}_{HL}$. According to equations (32), we get that

$$\tilde{\tau}_{LL} = 1 + \tilde{\tau}_{HL}, \tag{39}$$

$$\tilde{\tau}_{HL} = 1 + \frac{N-2}{2(N-1)}\tilde{\tau}_{LL}. \tag{40}$$

Solving the above equations, we have that $\tilde{\tau}_{LL} = 4(N-1)/N$ and $\tilde{\tau}_{HL} = (3N-4)/N$, which leads to $\tilde{\tau}^{(1)} = (3N-4)/N$. Based on these, we have that under pairwise comparison rules,

$$\sigma = \frac{N-2}{2(N-1)} \tag{41}$$

for star graphs.

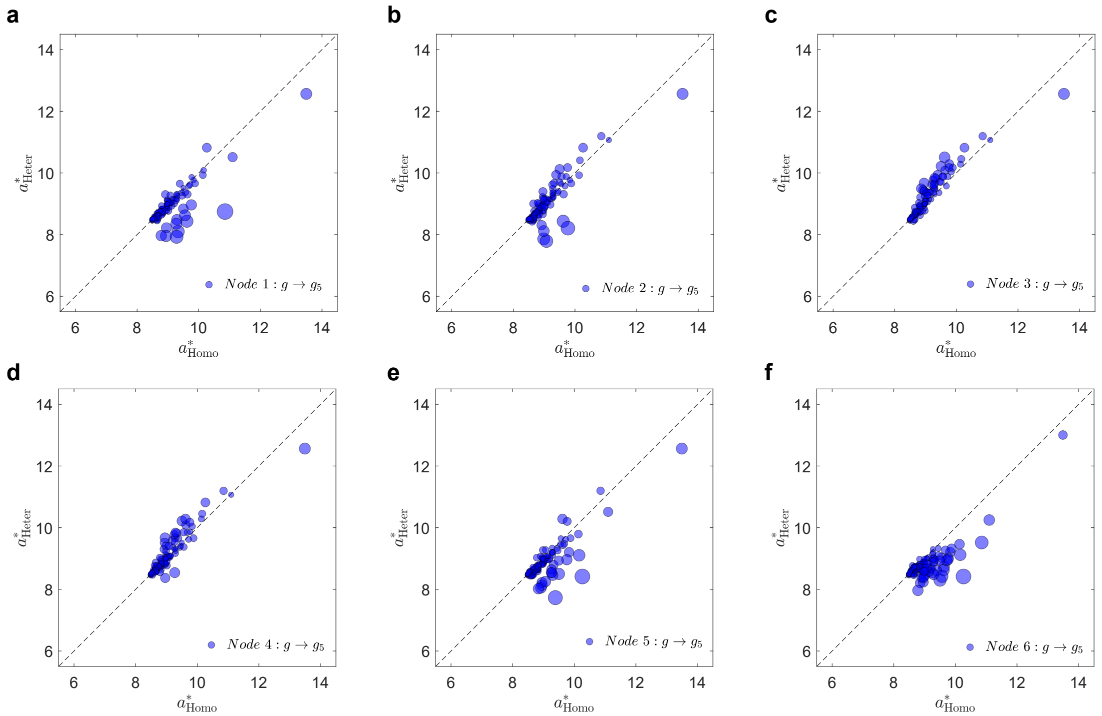

Supplementary Figure 1: Sensitivity of imitative dynamics to (minimal) heterogeneity of decision-making functions for all networks of size six. Here, the decision-making function $g(u) = 1/(1 + \exp(-u))$ and $g_5(u) = 1/(1 + 10\exp(-u))$. In each panel, the results are obtained under the situation where only one individual l ($l = 1, 2, \ldots,$ or 6) uses decision-making function $g_5(u)$ and all the rest use $g(u)$. In detail, $l = 1$ in panel (**a**), $l = 2$ in panel (**b**), $l = 3$ in panel (**c**), $l = 4$ in panel (**d**), $l = 5$ in panel (**e**), and $l = 6$ in panel (**f**). Other model settings are the same as those in Figure 4 of the main text.

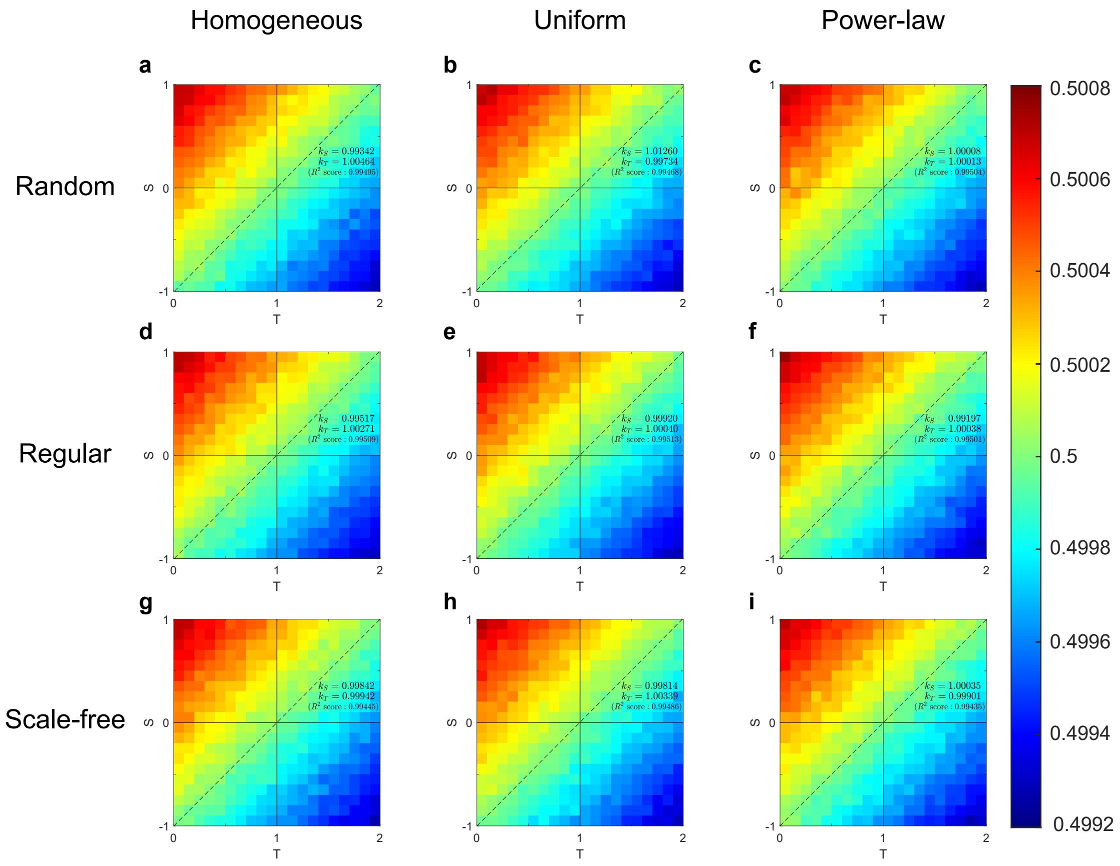

Supplementary Figure 2: Average abundance of strategy A in different social dilemmas. Here, we run simulations to calculate the average abundance of strategy A under the classical game payoff scheme with $a = R = 1$, $b = S$, $c = T$, and $d = P = 0$ where $-1 \leq S \leq 1$ and $0 \leq T \leq 2$. Different values of T and S represent different social dilemmas [16]: Stag-Hunt games ($1 > T > 0 > S$, lower left quadrant), Prisoner's Dilemma games ($T > 1 > 0 > S$, lower right quadrant), and Snowdrift games ($T > 1 > S > 0$, upper right quadrant). Similar to Figure 2 of the main text, we construct weighted networks by first generating an undirected network and then assigning weights to edges. In the figure, three kinds of unweighted networks (random, regular, and scale-free networks) and three types of edge weight distributions (homogeneous, uniform, and power-law distributions) are considered (see Figure 2 of the main text for the definitions of different edge weight distributions). This in total generates nine kinds of weighted networks. Each panel shows the simulation results under one kind of the weighed networks: **a** random networks with homogeneous weights, **b** random networks with uniform weights, **c** random networks with power-law weights, **d** regular networks with homogeneous weights, **e** regular networks with uniform weights, **f** regular networks with power-law weights, **g** scale-free networks with homogeneous weights, **h** scale-free networks with uniform weights, and **i** scale-free networks with power-law weights. In each panel, the color indicates the average abundance of strategy A, and the dashed line the estimated critical equation in the form of $1 + k_S S - k_T T = 0$ that corresponds to the condition for strategy success (inequality (3) in the main text). In detail, parameters k_S and k_T are normalized regression coefficients and R^2 score is the R-squared values, which are estimated by the linear regression with R, S, T, P as independent variables and the average abundance of strategy A minus one-half divided by the selection intensity as the dependent variable (see similar methods in Section *Estimating the coefficients via simulation* of the Supplementary Methods in [5] for detailed explanations). Other parameters and simulation settings are the same as those in Figure 2 of the main text.

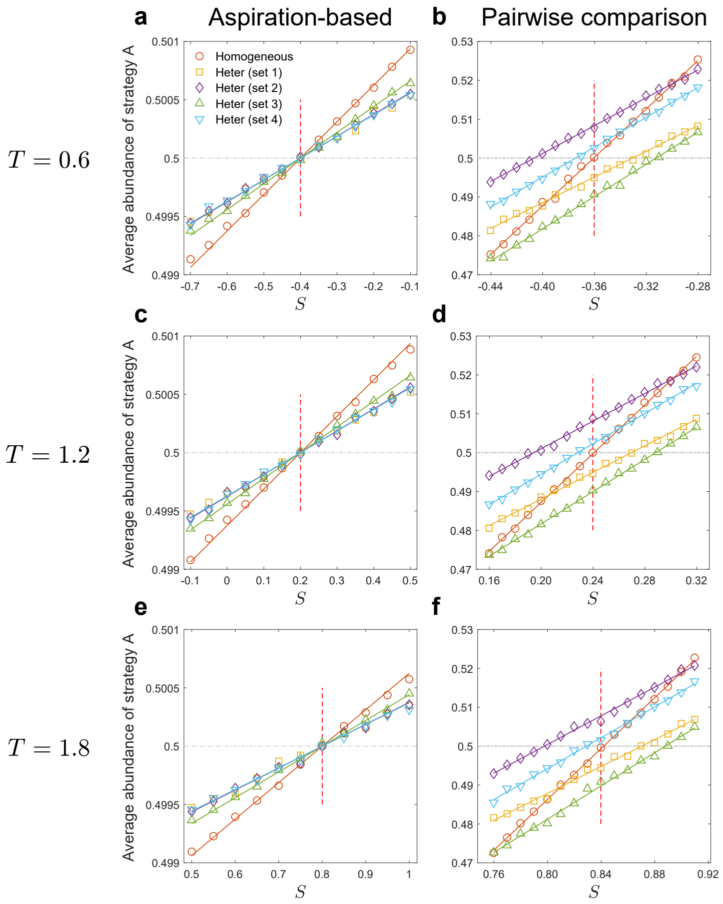

Supplementary Figure 3: Robustness of aspiration dynamics (panels (**a**), (**c**), and (**e**)) vs. sensitivity of imitative dynamics (panels (**b**), (**d**), and (**f**)) to heterogeneity of decision-making functions on scale-free networks with a power-law distribution of edge weights (network size $N = 100$). Here, we employ the classical game payoff scheme with $a = R = 1$, $b = S$, $c = T$, and $d = P = 0$ where $-1 \leq S \leq 1$ and $0 \leq T \leq 2$. To better illustrate the critical value of S above which $\langle x_A \rangle > \langle x_B \rangle$ and below which $\langle x_A \rangle < \langle x_B \rangle$, we fixed the value of T and leave S as a tunable parameter. Three values of T are considered: $T = 0.6$ (panels (**a**) and (**b**)), $T = 1.2$ (panels (**c**) and (**d**)), $T = 1.8$ (panels (**e**) and (**f**)). In each panel, we run simulations for five sets of update functions, one is homogeneous and the other four are heterogeneous. For the homogeneous case, we set the update functions of all individuals as $g_1(u) = 1/(1+\exp(-u))$. For the heterogeneous cases, we construct update functions by taking the convex combination of three base update functions, $g_1(u) = 1/(1+\exp(-u))$, $g_2(u) = 1/(1 + 10\exp(-u))$, and $g_3(u) = 10/(10 + \exp(-u))$. This means that update functions are now represented by $\gamma_1 g_1 + \gamma_2 g_2 + \gamma_3 g_3$ where $\gamma_1, \gamma_2, \gamma_3 \in [0, 1]$ and $\gamma_1 + \gamma_2 + \gamma_3 = 1$. Under such constraints, we then randomly sample $\gamma_1, \gamma_2, \gamma_3$ and use them to parameterize the update function for each individual. In the simulations, four sets of heterogeneous update functions are considered, which are denoted as Heter (set 1), Heter (set 2), Heter (set 3), and Heter (set 4) for short in the panels, respectively. In all the panels, symbols represent the simulation results and red dashed lines the critical value of S, S^*, determined by the condition for strategy success: $S^* = T - 1$ for aspiration-based update rules with both homogeneous and heterogeneous update functions (see inequality (3) in the main text for reference); $S^* = T - \frac{\tilde{\tau}^{(1)}-1}{\tilde{\tau}^{(1)}+1}$ for pairwise comparison update rules with homogeneous update functions (see Supplementary Note 3.3 for details). In addition, solid lines in panels (**a**), (**c**), and (**e**) represent the average abundance of strategy A determined by the analytical equation (16) in Supplementary Note 3.2.2; those in panels (**b**), (**d**), and (**f**) are obtained by a linear fit to the corresponding simulation results and are used to guide the eye. Other parameters: $\alpha_l = 2.0$ ($l = 1, 2, \cdots, N$), $\beta = 0.05$, and $\mu \to 0$ (see Supplementary Note 3.3 for details).

Supplementary References

[1] Allen, B. *et al.* Evolutionary dynamics on any population structure. *Nature* **544**, 227 (2017).

[2] Allen, B. & McAvoy, A. A mathematical formalism for natural selection with arbitrary spatial and genetic structure. *J. Math. Biol.* **78**, 1147–1210 (2019).

[3] Szabó, G. & Tőke, C. Evolutionary prisoner's dilemma game on a square lattice. *Phys. Rev. E* **58**, 69–73 (1998).

[4] Traulsen, A., Nowak, M. A. & Pacheco, J. M. Stochastic dynamics of invasion and fixation. *Phys. Rev. E* **74**, 011909 (2006).

[5] Wu, B. & Zhou, L. Individualised aspiration dynamics: Calculation by proofs. *PLoS Comput. Biol.* **14**, e1006035 (2018).

[6] Zhou, L., Wu, B., Vasconcelos, V. V. & Wang, L. Simple property of heterogeneous aspiration dynamics: Beyond weak selection. *Phys. Rev. E* **98**, 062124 (2018).

[7] Nowak, M. A., Sasaki, A., Taylor, C. & Fudenberg, D. Emergence of cooperation and evolutionary stability in finite populations. *Nature* **428**, 646–650 (2004).

[8] Tarnita, C. E., Ohtsuki, H., Antal, T., Fu, F. & Nowak, M. A. Strategy selection in structured populations. *J. Theor. Biol.* **259**, 570–581 (2009).

[9] Grinstead, C. M. & Snell, J. L. *Introduction to Probability* (American Mathematical Society, Providence, RI, 2012).

[10] Tarnita, C. E. & Taylor, P. D. Measures of relative fitness of social behaviors in finite structured population models. *Am. Nat.* **184**, 477–488 (2014).

[11] Nowak, M. A., Tarnita, C. E. & Antal, T. Evolutionary dynamics in structured populations. *Phil. Trans. R. Soc. B* **365**, 19–30 (2010).

[12] Allen, B. & Nowak, M. A. Games on graphs. *EMS Surv. Math. Sci.* **1**, 113–151 (2014).

[13] Ohtsuki, H., Hauert, C., Lieberman, E. & Nowak, M. A. A simple rule for the evolution of cooperation on graphs and social networks. *Nature* **441**, 502–505 (2006).

[14] Vasconcelos, V. V., Santos, F. P., Santos, F. C. & Pacheco, J. M. Stochastic dynamics through hierarchically embedded markov chains. *Phys. Rev. Lett.* **118**, 058301 (2017).

[15] Van Cleve, J. Social evolution and genetic interactions in the short and long term. *Theor. Popul. Biol.* **103**, 2–26 (2015).

[16] Santos, F. C., Pacheco, J. M. & Lenaerts, T. Evolutionary dynamics of social dilemmas in structured heterogeneous populations. *Proc. Natl. Acad. Sci. USA* **103**, 3490–3494 (2006).
