# Supplementary material for: Aspiration dynamics generate robust predictions in heterogeneous populations
Source: Nat Commun. 2021 May 31;12:3250. doi: 10.1038/s41467-021-23548-4 (PMC8166829; doi:10.1038/s41467-021-23548-4)
Supplement: Supplementary file 2 — Reporting Summary [file 41467_2021_23548_MOESM2_ESM.pdf]

nature research

Corresponding author(s): Lei Zhou

Last updated by author(s): Apr 30, 2021

Reporting Summary

Nature Research wishes to improve the reproducibility of the work that we publish. This form provides structure for consistency and transparency in reporting. For further information on Nature Research policies, see our Editorial Policies and the Editorial Policy Checklist.

Statistics

For all statistical analyses, confirm that the following items are present in the figure legend, table legend, main text, or Methods section.

n/a	Confirmed	
☐	☒	The exact sample size (*n*) for each experimental group/condition, given as a discrete number and unit of measurement
☒	☐	A statement on whether measurements were taken from distinct samples or whether the same sample was measured repeatedly
☒	☐	The statistical test(s) used AND whether they are one- or two-sided<br>*Only common tests should be described solely by name; describe more complex techniques in the Methods section.*
☒	☐	A description of all covariates tested
☒	☐	A description of any assumptions or corrections, such as tests of normality and adjustment for multiple comparisons
☐	☒	A full description of the statistical parameters including central tendency (e.g. means) or other basic estimates (e.g. regression coefficient) AND variation (e.g. standard deviation) or associated estimates of uncertainty (e.g. confidence intervals)
☒	☐	For null hypothesis testing, the test statistic (e.g. *F*, *t*, *r*) with confidence intervals, effect sizes, degrees of freedom and *P* value noted<br>*Give P values as exact values whenever suitable.*
☒	☐	For Bayesian analysis, information on the choice of priors and Markov chain Monte Carlo settings
☒	☐	For hierarchical and complex designs, identification of the appropriate level for tests and full reporting of outcomes
☒	☐	Estimates of effect sizes (e.g. Cohen's *d*, Pearson's *r*), indicating how they were calculated

Our web collection on statistics for biologists contains articles on many of the points above.

Software and code

Policy information about availability of computer code

Data collection	Data was generated using custom Fortran code, written with the software Microsoft Visual Studio Community 2019. The custom Fortran code we use is made available in Zenodo with the identifier https://doi.org/10.5281/zenodo.4723407.
Data analysis	Data was analyzed using MATLAB version R2020a. We use Microsoft Visual Studio Community 2019 to write custom Fortran code.

For manuscripts utilizing custom algorithms or software that are central to the research but not yet described in published literature, software must be made available to editors and reviewers. We strongly encourage code deposition in a community repository (e.g. GitHub). See the Nature Research guidelines for submitting code & software for further information.

Data

Policy information about availability of data

All manuscripts must include a data availability statement. This statement should provide the following information, where applicable:

- Accession codes, unique identifiers, or web links for publicly available datasets
- A list of figures that have associated raw data
- A description of any restrictions on data availability

All data generated or analyzed during this study are included within the paper and its supplementary information files.

Field-specific reporting

Ecological, evolutionary & environmental sciences study design

All studies must disclose on these points even when the disclosure is negative.

Study description	We studied the condition for strategy success in evolutionary games on weighted networks. Most of our results are based on mathematical derivations and proofs (see details in the Supplementary Information). We also run Monte Carlo simulations to verify our theoretical results using Fortran.
Research sample	Numerical data was generated using custom Fortran code, which we make available in Zenodo with the identifier https://doi.org/10.5281/zenodo.4723407.
Sampling strategy	For the calculation of the average abundance of strategy A in Figure 2, we obtain one data point by averaging the average abundance of strategy A over 200 runs; in each run, we set the total number of simulation time steps to be 2*10^7 with a relaxation time of 1*10^7 (this leads to a sample size of 1*10^7) to ensure that enough information is obtained for the stationary distribution.
Data collection	Data was generated using the Fortran code.
Timing and spatial scale	Not applicable
Data exclusions	No
Reproducibility	All numerical data can be reproduced using the Fortran code we provide.
Randomization	Not applicable
Blinding	No blinding was needed for the computer-generated data.

Did the study involve field work? ☐ Yes ☒ No

Reporting for specific materials, systems and methods

We require information from authors about some types of materials, experimental systems and methods used in many studies. Here, indicate whether each material, system or method listed is relevant to your study. If you are not sure if a list item applies to your research, read the appropriate section before selecting a response.

Materials & experimental systems

n/a	Involved in the study	
☒	☐	Antibodies
☒	☐	Eukaryotic cell lines
☒	☐	Palaeontology and archaeology
☒	☐	Animals and other organisms
☒	☐	Human research participants
☒	☐	Clinical data
☒	☐	Dual use research of concern

Methods

n/a	Involved in the study	
☒	☐	ChIP-seq
☒	☐	Flow cytometry
☒	☐	MRI-based neuroimaging
